# A Review on the Protecting Effects and Molecular Mechanisms of Berries Against a Silent Public Health Concern: Non-Alcoholic Fatty Liver Disease

**DOI:** 10.3390/antiox13111389

**Published:** 2024-11-14

**Authors:** Anshul Sharma, Hae-Jeung Lee

**Affiliations:** 1 Department of Food and Nutrition, College of Bio Nano Technology, Gachon University, Seongnam-si 13120, Gyeonggi-do, Republic of Korea; anshulsharma@gachon.ac.kr; 2 Institute for Aging and Clinical Nutrition Research, Gachon University, Seongnam-si 13120, Gyeonggi-do, Republic of Korea; 3 Department of Health Sciences and Technology, GAIHST, Gachon University, Incheon 21999, Republic of Korea

**Keywords:** berries, oxidative stress, steatosis, gut microbiota, reactive oxygen species, inflammation

## Abstract

Non-alcoholic fatty liver disease (NAFLD) poses a silent threat to human health, with prevalence rising at an alarming rate. The treatment and prevention of NAFLD depend on novel approaches as no effective treatment options are currently available. Berries are unique sources of phenolic compounds that have proven roles in disease prevention and health promotion. However, a comprehensive review of the effects of different berries on NAFLD and related pathologies is lacking. Thus, the present review aims to summarize the effects of berry extracts, plant parts, and bioactive compounds from twenty-one different berries on NAFLD. The molecular mechanisms involved include the regulation of lipid homeostasis, modulation of oxidative stress and inflammation markers, and activation of different signaling pathways in different in vitro and in vivo NAFLD models. Furthermore, their modulatory effects on the gut microbiota have also been highlighted. Clinical intervention research on the benefits of berries in NAFLD is limited; nonetheless, this paper discusses clinical studies demonstrating the effects of different berries in people with NAFLD. Future research should focus on long-term clinical studies to compare the therapeutic potentials of different berries against NAFLD.

## 1. Introduction

The prevalence of liver diseases is increasing globally. More than two million people die each year owing to complications from chronic liver disease. The primary risk factors in the development of liver disease are alcoholism, hepatitis B and C, obesity, and metabolic syndrome. Non-alcoholic fatty liver disease (NAFLD) and alcoholic fatty liver (AFL) have no effective treatments available; however, viral hepatitis can be recovered from [1,2,3]. NAFLD is one of the main causes of chronic liver disease worldwide, accounting for the majority of liver-related morbidity and mortality. The global prevalence of NAFLD has increased by approximately 30.05% [2]. The highest (44.37%) and lowest (25.10%) incidence rates were reported in Latin America and Western Europe, respectively [2,4]. The prevalence rates in Korean men and women were reported to be 38.3% and 12.6%, respectively, with an overall incidence of 27.3% [5]. Its prevalence rate in Japan is 25.5%, with a higher incidence observed in males and notable regional variations [6]. In 2018, the prevalence of NAFLD in China escalated to 32.9% [7]. In 2020, NAFLD was renamed metabolic dysfunction-associated fatty liver disease (MAFLD) [8,9,10]. 

**Figure 1:**
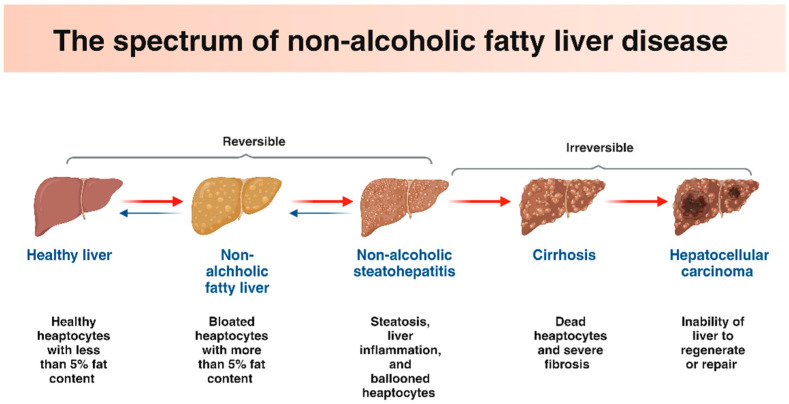
Spectrum of non-alcoholic fatty liver disease. The illustration depicts the transition from a healthy liver to non-alcoholic fatty liver, which then progresses to non-alcoholic steatohepatitis. The advancement of the disease results in irreversible cirrhosis and hepatocellular cancer. Image created using BioRender (www.biorender.com), assessed on 15 October 2024.

A balance between lipid anabolic and catabolic events, which regulate lipid levels in hepatic cells, allows for the maintenance of lipid homeostasis in cells [11]. This delicate balance shifts out of equilibrium within liver cells, resulting in metabolic illnesses, including NAFLD [11]. The term NAFLD, which was first used to describe the condition in the 1980s, is a multisystem disease that covers a wide histological continuum, from excessive fat deposits in the liver (steatosis) to more severe clinical manifestation non-alcoholic steatohepatitis (NASH), which is characterized by hepatic inflammation (steatohepatitis), which leads to the scarring of liver tissue (fibrosis), and the last stage could be hepatocellular carcinoma (HCC) [12]. Hepatic steatosis denotes the initial recoverable phase of NAFLD, whereas NASH indicates an inflammatory condition of the liver characterized by persistent oxidative stress and impairment [13] (Figure 1). The treatment of NAFLD and its associated comorbidities, such as insulin resistance (IR), cardiovascular disease, and diabetic nephropathy, are currently being researched to target hepatic lipid metabolism [14].

NASH is characterized by hepatic lipid accumulation in over 5% of the liver weight without considerable alcohol intake, accompanied by histological characteristics such as liver injury, lobular inflammation, and fibrosis. Some researchers described NASH pathogenesis as a “two-hit” theory, in which the first one usually arises from metabolic complications of obesity, IR, diabetes mellitus, and metabolic syndrome (MetS). The second hit comprises oxidative stress, inflammation, the immune system, adipose tissue-derived factors, gut-derived factors, and hereditary factors and promotes the evolution of NASH, causing liver damage and fibrosis [15]. The simultaneous persistence of many metabolic disorders may play a crucial role in producing subsequent pathological characteristics in the natural course of NASH, as opposed to a single risk factor. 

However, the multiple-hit pathogenesis theory advocates the participation of several factors, such as diet, IR, adipose hormones, and genetic and epigenetic factors, which interact concurrently or sequentially in genetically predisposed individuals, leading to varying stages of NAFLD [16]. Lysosomal acid lipase (LAL) is often linked to lipid homeostasis and is currently under investigation for its involvement in NAFLD development. LAL can act as a potential noninvasive diagnostic biomarker because its reduced activity is related to NAFLD pathogenesis [17]. 

The pertinent signaling pathways and molecular mechanisms underlying the transition from NAFLD to NASH remain unknown. This knowledge gap severely restricts the creation of NASH-specific drugs, and until 2023, no recognized treatments for NASH other than lifestyle changes (nondrug therapy), including diet and exercise, have been reported [18,19]. Nonetheless, in 2024, the Food and Drug Administration approved resmetirom for the treatment of NASH in adults in conjunction with diet and exercise, and is currently being reviewed in the EU for the treatment of NASH [20,21]. Furthermore, little information is available on non-surgical biomarkers that could assist in the diagnosis, stratification of risk, and follow-up of patients. Approved remedies and dependable disease-specific preclinical models to evaluate the effect of drugs and biomarker efficacy and precision are lacking [22]. These factors necessitate continued efforts to identify drugs, including those from natural sources, as effective therapeutics against NAFLD. 

## 2. NAFLD Pathogenesis

Despite an abundance of available studies on NAFLD, its etiology and pathophysiology remain unclear. Numerous studies have demonstrated that NAFLD is caused by multiple factors. According to the “two-hit” hypothesis, oxidative stress is a key element in producing liver damage. Increased reactive oxygen species (ROS) damage the oxidative phosphorylation pathway, disrupt uncoupling, and harm cell membranes, exacerbating liver inflammation. When the liver retains a large amount of fat, oxidative stress increases dramatically, leading to the generation of excessive fatty acids for energy. Persistent oxidative stress stimulates the liver, potentially resulting in NASH and cirrhosis [23]. The other factor is IR. In IR, the response of the body to insulin decreases owing to various factors. To sustain a steady blood glucose level, the body secretes additional insulin as compensation, which leads to hyperinsulinemia. IR causes an elevation in free fatty acids (FFA) because of its effect on lipid metabolism, leading to an extreme buildup of fatty acids in the liver. Moreover, the secretion of very low-density lipoproteins is diminished, resulting in elevated triglyceride (TG) levels [24]. Mitochondria plays crucial roles in energy production, calcium balance, lipid metabolism, and modulation [25]. The growing body of research indicates that mitochondrial dysfunction is a notable catalyst for NAFLD and its progression, with several mechanisms potentially involved, including lipid peroxidation and oxidative stress, energy metabolism disorders, anomalies related to the metabolism of fatty acids, anomalous mitophagy, and hepatocellular apoptosis mediated by mitochondria [26,27,28]. Thus, approaches to prevent mitochondrial damage or modulate mitochondrial function in a clinically beneficial manner may provide effective remedies to treat NAFLD.

## 3. Risk Factors

**Figure 2:**
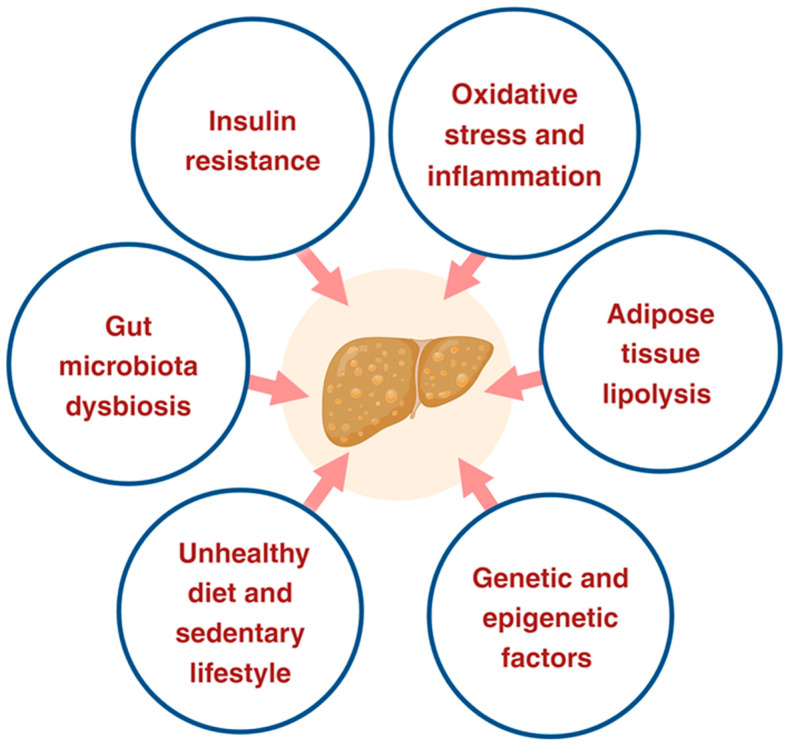
Risk factors of non-alcoholic fatty liver disease (NAFLD). NAFLD is a complex and multifaceted disease and its progression is influenced by various factors, including unhealthy diet, sedentary lifestyle, oxidative stress and inflammation, gut microbiota dysbiosis, insulin resistance, adipose tissue lipolysis, and genetic and epigenetic factors. Image created using BioRender (www.biorender.com), assessed on 15 October 2024.

Various factors increase the risk and progression of NASH. These factors include poor diet and nutrition, oxidative stress and inflammation, sedentary lifestyle, genetic and epigenetic factors, and gut microbiota dysbiosis [29] (Figure 2). The presence of MetS, obesity, IR, lifestyle, socioeconomic status, age, race, sex, and pediatric and adolescent NAFLD are prominent risk factors for NAFLD and NASH [3,30,31]. Each of these factors is thought to facilitate lipid accumulation inside hepatocytes, thereby advancing the progression of NAFL to NASH, and subsequently to NASH-related cirrhosis or HCC [32]. Although obesity is a primary risk factor for the development of NAFLD, non-obese individuals and those without MetS components are susceptible to NAFLD [33]. Multiple pathophysiological mechanisms responsible for NAFLD progression have been individually evaluated, resulting in new knowledge about its pathological characteristics. Changes in the gut microbiota have been associated with hepatic inflammatory signaling, which facilitates IR, hepatic steatosis, and non-alcoholic steatohepatitis. Sleep apnea and vitamin D deficiency are also considered risk factors for NAFLD. 

## 4. Methodology for Literature Review 

A thorough literature search was performed using easily accessible sources, such as Embase, PubMed, Google Scholar, and Web of Science. The repositories were searched for keywords: “NAFLD”, or “berries and NAFLD”, or “Goji berry”, or “bayberry”, or “Berberry”, or “Blueberry”, or “Chokeberry”, “Gooseberry”, or “Bilberry”, or “Blackberry”, or “Açai berry”, “Blackcurrant”, or “Acerola”, or “Cranberry”, or “Mangosteen”, or “Lingonberry”, or “Maoberry”, or “Maqui”, or “Raspberry”, or “Honeyberry”, or “Saskatoon berry”, or “Seabuckthorn”, or “Schisandra berry”, and “berries with their scientific names”. These keywords were used to find all the published in vitro, in vivo, and clinical studies. This paper consists of 177 studies, of which 56 studies represent data from cells and different animal models and 13 studies represent clinical trials, including three meta-analyses and one electronic preprint. Research papers published between 2011 and 2024 are included in this review. Abstracts and manuscripts written in languages other than English were excluded from the analysis. 

## 5. Protecting Effects of Berries 

Over the past decade, the use of phytotherapy to mitigate health risks and enhance human health has markedly increased [34]. Numerous promising pharmacological interventions for NASH are currently undergoing clinical trials and are expected to facilitate the development of novel and efficacious therapeutic alternatives [35]. Berries are well known for their antioxidant activity owing to the presence of bioactive components, especially anthocyanins. Many reviews detailing the nutrients and phenolic constituents, along with their health-promoting activities, have been published. The protective effects of berries include oxidative stress suppression, anti-inflammatory activities, anti-obesity and anti-diabetic properties, neuroprotective effects, protection against cardiovascular diseases, antimicrobial benefits, and support against ulcerative colitis [36,37,38]. Moreover, the modulatory effects of berries on human gut microbiota have also been identified [39]. In this direction, different berries rich in antioxidants have been utilized by many researchers globally as therapeutic options for NAFLD. To the best of our knowledge, dedicated reviews focusing on the protective effects of berries, plant parts, and various bioactive components against NAFLD and related pathologies are lacking. Thus, this review provides comprehensive information on the effects of 21 different berries on NAFLD markers. The following sections describe the ethnobotanical aspects of different berries and their therapeutic efficacies in in vitro and in vivo NAFLD models. In addition, to date, limited clinical studies have been conducted. Table 1 describes the effects of different berries on in vitro and in vivo NAFLD models. 

### 5.1. Goji Berry

Goji berries (*Lycium barbarum*) have grown in popularity in recent years because they are widely recognized as a “super food” with exceptionally excellent nutritional and antioxidant properties. Goji berries, commonly known as wolfberries, are traditional Chinese medicinal herbs belonging to the Solanaceae family. The different plant parts (stems, leaves, roots, and fruits) have both nutritional and medicinal uses [40]. Various bioactive components in goji berries are responsible for their benefits [41]. 

Among them, polysaccharides (LBP) are acknowledged as important precursors responsible for a variety of biological activities, including glucose regulation in diabetes, neuroprotection, aging, antioxidant characteristics, anti-fatigue/endurance, immunomodulation, enhanced metabolism, protection against glaucoma, and antitumor activity [42]. In one study, aqueous and ethanolic extracts of *L. barbarum* (LBAE, LBEE) showed protective effects against high-fat diet (HFD)-induced oxidative stress in Wistar rats by reducing hepatic damage and oxidative changes and by normalizing lipid and antioxidant levels in animal models [43]. Xiao et al. [44] explored the protective effects of LBP (at a dose of 1 mg/kg) against the induction of NASH in a voluntary orally fed HFD rat model. The aberrant liver characteristics caused by NASH were notably improved by co-treatment with LBP. The preventive effects, such as relieved oxidative stress, improved histology and FFA levels, rebalancing of lipid metabolism, and reduction in the production of various proinflammatory cytokines, were found to be related to the modulation of several molecular pathways, including phosphoinositide 3-kinase (PI3K)/serine/threonine kinase (Akt)/forkhead box protein O1, c-Jun N-terminal kinase (JNK)/c-Jun, and liver kinase B1 (LKB1)/5′ AMP-activated protein kinase (AMPK) and their downstream targets [44] (Table 1). Furthermore, the impaired autophagy also contributes to NAFLD. Xiao et al. [45] used LBP to demonstrate their therapeutic efficacy in curing steatosis in both cell and animal models. LBP supplementation reduce steatosis by decreasing sterol regulatory element binding protein 1c (SREBP-1c) expression and controlling inflammatory cytokines. This was accomplished in part by limiting the activation of the nuclear factor kappa B (NF-κB) pathway. Additionally, improvements were observed in hepatic damage, IR, and oxidative stress. Autophagy has been suggested to reduce oxidative damage and IR. In vitro studies showed that the favorable properties of LBP partially attributed to the functions of β-carotene and l-arabinose. The authors hypothesized that LBP ameliorate NAFLD/NASH through various pathways, including autophagy [45]. 

**Figure 3:**
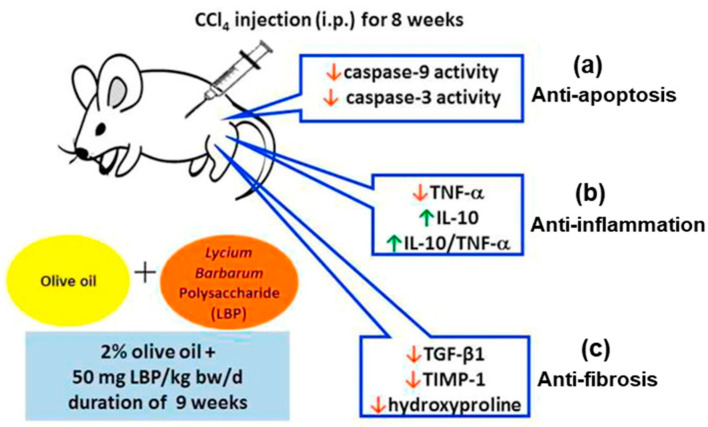
The protective effects of olive oil and *Lycium barbarum* polysaccharides (LBP) in CCl_4_-injected mice. The treatment resulted in (a) anti-apoptosis, (b) anti-inflammatory and (c) anti-fibrosis effects. CCl_4_: carbon tetrachloride, TIMP-1: tissue inhibitor of metalloproteinase-1, TGF-β1: transforming growth factor-beta 1, IL-10: interleukin-10, TNF-α: tumor necrosis factor alpha. ↑: increase, ↓: decrease. Reproduced (with modifications) with kind permission from Ref. [47]. Copyright 2018 Elsevier.

Another study has confirmed the crucial role of LBP in reducing HFD-induced hepatic steatosis in both cellular and animal models. A new functional relationship between LBP and the sirtuin1 (SIRT1)/AMPK pathway in hepatic lipid metabolism was revealed by the antisteatotic effect of LBP, which was linked to the upregulation of SIRT1 deacetylase activity and expression [46]. A recent study revealed that supplementation of 50 mg LBP/kg BW/day for nine weeks improved liver inflammatory, fibrotic, and apoptotic markers in rats with liver fibrosis stimulated by an intraperitoneal injection of carbon tetrachloride (CCl_4_). Hepatic injury by CCl_4_ exposure reported to be caused by free radical generation. According to the authors, the combined treatment of olive oil (pretreatment) with LBP was more effective against liver fibrosis, demonstrating anti-inflammatory and anti-apoptotic effects [47] (Table 1) (Figure 3). 

Hepatic injuries caused by methionine and choline-deficient (MCD) diet-induced NASH in C57BL/6N mice, including steatosis, fibrosis, inflammation, and apoptosis, were reduced by LBP treatment. The hepatoprotective effects of LBP are partially attributed to the suppression of NF-κB activity and nod-like receptor protein 3/6 assembly [48]. As stated above, the berries have the ability to modulate an unbalanced gut microbiota. In this line, the effect of goji berry when combined with aerobic exercise was discovered to ameliorate NAFLD in HFD-fed rats by augmenting short-chain fatty acids (SCFAs) and promoting the growth of Bacteroidetes while reducing Firmicutes/Bacteroidetes ratio and levels of Proteobacteria. This study demonstrated the prebiotic effects of LBP combined with aerobic exercise [49] (Table 1). These studies suggest that LBP plays an important role in the regulation of hepatic lipid metabolism and thus may contribute to the expansion of a new beneficial drug for NAFLD. 

#### Clinical Study 

In a previous study, the prebiotic potential of LBP from goji berries was shown to stimulate the growth of beneficial bacteria and restore gut dysbiosis. In this study, a randomized, double-blind, placebo-controlled clinical trial (RCT) was conducted to evaluate the therapeutic prebiotic potential of LBP capsules (at 40% concentration) in 50 participants aged 45–59 years over a study period of 3 months. Compared to the placebo (*n* = 25), LBP supplementation relieved alanine transaminase (ALT) concentration and modulated gut dysbiosis in patients with NAFLD (*n* = 25) [50]. This study suffers from the limitation that the prebiotic potential of LBP cannot be generalized. This study demonstrated the beneficial effects of LBP in humans. However, better-designed, high-quality clinical studies are warranted to ascertain the full potential of these berries. 

### 5.2. Bayberry 

Bayberry (*Myrica*) is an evergreen fruit tree that is indigenous to Southeast Asia and China. The extraordinary antioxidant capacity of bayberry in vitro has been ascribed to its anthocyanins and diverse phenolic acids, including ferulic, caffeic, salicylic, and sinapic acid [51,52]. Other beneficial properties of bayberry include anticancer, anti-diabetic, anti-obesity, anti-inflammatory, anti-aging, and neuroprotective properties [53]. 

#### Clinical Study

Guo et al. [54] examined the effects of bayberry on 88 patients with NAFLD (body mass index (BMI) > 23.1 kg/m^2^) in a RCT crossover trial [54]. Eligible individuals were recruited and provided with 250 mL of bayberry juice or placebo twice daily for 4 weeks. At the end of the study, no significant differences were observed between the groups in terms of anthropometric parameters, including plasma TG, total cholesterol (TC), low-density lipoprotein cholesterol (LDL-C), fasting glucose, insulin, homeostatic model assessment for insulin resistance (HOMA-IR), ALT, aspartate aminotransferase (AST), and high-sensitivity C-reactive protein (hs-CRP). However, bayberry juice supplementation exhibited beneficial effects against markers related to oxidative stress, inflammation, and apoptosis. Compared to the control, the levels of tumor necrosis factor (TNF)-α, protein carbonyl groups (a serologic marker of oxidative stress), cytokeratin-18 (CK-18) fragment M30 (an apoptosis marker), interleukin (IL)-8, and tissue polypeptide-specific antigen (an apoptosis marker) were significantly reduced in the treatment subjects. CK-18 is a keratin, which is considered an important substrate of caspases during hepatocyte apoptosis [55]. The authors have highlighted the most important findings regarding CK-18 reduction. No adverse events were observed. To the best of our knowledge, this is the first study to use a crossover design. However, one of the limitations of this study was the lack of assessment regarding the statistical power and the necessity of the sample size. Furthermore, study blinding was not fully implemented because of the taste differences between the treatment and placebo groups. Moreover, an accurate evaluation of polyphenol intake was carried out before and after the intervention because participants were asked not to consume polyphenol-rich foods throughout the study period. Determining the effective polyphenol intake from dietary records is fundamentally difficult because of the significant diversity in polyphenol content among different vegetables and fruits, as well as within certain species based on maturity [54]. 

### 5.3. Berberry 

Berberries are derived from *Berberis vulgaris* L., a member of the family Berberidaceae. Berberine is an organic compound obtained from herbs found in India and China. *Berberis* exhibits various health-promoting properties, including antioxidant, anti-inflammatory, hepatoprotective, antimicrobial, and anti-diabetic activities [56]. 

#### Clinical Study

An RCT was performed on patients with MetS (*n* = 12), supplemented with capsules (500 mg/capsule) of berberine trichloride three times each day before a meal for 90 days. The results showed that daily intake of berberine capsules was capable of reducing MetS with a decrease in waist circumference, systolic blood pressure, TG, and insulin secretion, with an improvement in insulin sensitivity [57]. These effects may be explained by the modification of adipogenesis, glycolysis, and insulin secretion, which may inhibit mitochondrial dysfunction, activate the AMPK pathway, and increase glucose transporter type 4 and glucagon-like peptide-1 [58]. Supplementation with berberine also inhibited the angiotensin-converting enzyme, and release of nitric oxide by activating cyclic guanosine monophosphate, and acted as an antagonist of alpha one-adrenoreceptors on the vasculature. The study reported no adverse effects. However, the present study did not assess the effects of berberine on the inflammatory markers. Future studies should include these experiments. Long-term studies with larger sample sizes are warranted in this direction. 

In another study, berberine capsules (0.5 g) reduced hepatic fat levels, possibly via diminishing circulating serum levels of ceramide-1-phosphate and ceramide in 41 patients with NAFLD after 16 weeks of treatment. Liquid chromatography–mass spectrometry was used to evaluate the serum lipid metabolites in patients before and after berberine treatment. Notably, the study reported that berberine has a specific effect on sphingolipids, including a reduction in ceramides, which play a key role in the pathogenesis of NAFLD. However, lifestyle modification alone had little influence on ceramide levels. The limitation of this study is attributed to its cross-sectional design and the inability to perform liver biopsy on patients because of ethical constraints. Thus, additional research is necessary to investigate the effect of berberine capsules on the human hepatic lipid profile and associated lipid metabolism genes [59]. In another randomized, parallel-controlled, open-label clinical study, patients were provided lifestyle intervention (LSI), LSI and 15 mg of pioglitazone qd, or LSI and berberine (BBR) for 16 weeks. This study assessed the hepatic fat content, liver enzymes, serum glucose levels, serum and urine BBR concentrations, and serum lipid profiles, before and after supplementation. Intake of LSI with BBR resulted in a marked reduction in hepatic fat content and improvements in body weight, lipid profiles, and HOMA-IR compared with that of LSI alone. The authors indicated that BBR was absorbed following oral administration in the study participants. BBR has mild adverse effects on the digestive system. The study limitation was that no assessment was performed using liver biopsy owing to ethical concerns. The study also did not evaluate the effects of BBR on inflammatory markers, genes related to fibrosis, or energy metabolism [60].

Mechanistically, one study reported that BBR reduced TG accumulation in the liver and downregulated the expression of hepatic stearyl-coenzyme A desaturase 1 and genes involved in the production of TG [61]. BBR supplementation has been reported to recruit and trigger brown adipose tissue in both humans and mice [62]. In a recent study, 42 patients with NAFLD received 750 mg capsules of *B. integerrima* hydroalcoholic extract every 12 h for two months. Compared to the placebo, *Berberis* capsule supplementation resulted in considerably higher total antioxidant capacity, glutathione peroxidase levels, and high-density lipoprotein cholesterol (HDL-C) levels. Significantly lower levels of body mass index (BMI), TG, LDL-C, cholesterol, fasting blood glucose, AST, ALT, and alkaline phosphatase were observed. The effects of *Berberis* on inflammatory markers have not been evaluated. Cell line studies are required to evaluate the effects of *Berberis* on NAFLD [63]. In a recent RCT study, 100 subjects with NAFLD and diabetes were supplemented with berberine ursodeoxycholate, with 33 subjects in the high-dose group (1000 mg), 34 in the low-dose group (500 mg), and 33 in the placebo group, for a duration of 18 weeks. Subjects supplemented with the higher dose exhibited a significant reduction in liver fat content with improved glycemic control, liver enzymes, and weight loss. Diarrhea and abdominal distress were the most common adverse effects. Generally, low doses have been reported to be less effective [64]. 

A meta-analysis involving six RCTs and 501 patients with NAFLD confirmed that berberine improved lipid parameters, IR, hepatic markers, and the degree of hepatic steatosis. According to the meta-analysis, berberine could be a good choice for the treatment of NAFLD; however, the study included only six RCTs and did not assess the risk of publication bias owing to the limited number of studies. Furthermore, the longest study duration among the included studies was 16 weeks. The small sample size of most studies and lack of information on methods of blinding and allocation concealment could be considered as potential biases [65]. In another meta-analysis that included 27 RCTs and 2569 participants, berberine reduced TG and LDL-C levels and increased HDL cholesterol levels [66]. Hence, well-designed, large-scale, and long-term duration RCTs are warranted. In summary, these findings suggest that berberine protects against NAFLD by ameliorating oxidative stress, dyslipidemia, and diabetes. 

### 5.4. Blueberry 

Blueberry is a perennial flowering plant belonging to *Vaccinium* spp. of the family Ericaceae. Blueberry pomace and juice extracts have been documented to have antioxidant, hypoglycemic, hepatoprotective, and anti-obesity properties [67,68].

Ren et al. [69] reported that supplementation with blueberry juice and probiotics (BP) successfully protected the liver function in NAFLD. The protective effects were demonstrated by regulating a peroxisome proliferator-activated receptor (PPAR)-α-mediated SREBP-1c/patatin-like phospholipase domain-containing protein 3 (PNPLA-3) pathway [69]. In a subsequent study, the research group evaluated the effect of this combination on mitochondrial dysfunction and discovered that it effectively protected rats by modulating SIRT1 expression. Moreover, the treatment restored the mitochondrial respiratory system. Thus, authors proved that blueberry juice and probiotic tablets improved NAFLD in Sprague Dawley (SD) rats through SIRT1/PPARγ coactivator-1α (PGC-1α) [70].

**Figure 4:**
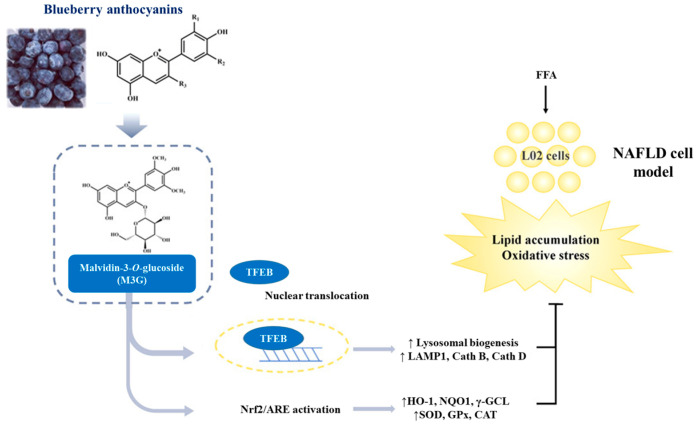
Schematic diagram illustrating how blueberry anthocyanin (M3G) inhibited lipid accumulation and oxidative stress in FFA-induced L02 cells (In vitro NAFLD model). FFA: free fatty acid, M3G: malvidin-3-*O*-glucoside, TFEB: transcription factor EB, LAMP1: lysosome-associated membrane protein-1, HO-1: heme oxygenase-1, γ-GCL: gamma-glutamyl cysteine ligase, SOD: superoxide dismutase, Nrf2: nuclear factor erythroid 2-related factor 2, Cath: cathepsin, GPx: glutathione peroxidase, ARE: antioxidant responsive element, CAT: catalase. ↑: increase. Reproduced (with modifications) with kind permission from Ref. [72]. Copyright 2021 American Chemical Society.

Using a rat NAFLD model, Zhu et al. [71] found that IL-22 participates in blueberry combined with probiotic therapy by activating the Janus kinase 1 (JAK1)/signal transducer and activator of transcription 3 (STAT3) signaling pathway and inhibiting the apoptotic factor Bcl-2-associated X protein (Bax). In the presence of IL-22, blueberry extract, together with probiotics, significantly decreased the accumulation of liver fat droplets and TG, whereas the opposite occurred in its absence. This study indicated that IL-22 is an important target, and its regulation is essential for blueberry-stimulated NAFLD therapy [71]. Considering the active candidate in blueberry and the underlying molecular mechanisms, using a FAA-induced steatosis in vitro (L02 cells) model, Xu et al. demonstrated that the anthocyanin malvidin-3-*O*-glucoside is the active constituent that ameliorates NAFLD by modulating the transcription factor EB (TFEB, a principal regulator of lysosome genesis)-mediated lysosomal function and activating the nuclear factor erythroid 2-related factor 2 (Nrf2)/antioxidant responsive element (ARE) signaling pathway, mitigating the redox state [72] (Table 1) (Figure 4). 

These studies indicate that blueberry components, along with probiotics, could be an effective treatment strategy for NAFLD. Further research is required to assess its effects on gut microbiota modulation using in vitro and in vivo models. Long-term clinical studies should be conducted to determine the therapeutic effects of blueberry supplemented with probiotics on NAFLD. 

### 5.5. Chokeberry

*Aronia melanocarpa*, also known as chokeberry, is a shrub of the Rosaceae family that is consumed as a native fruit in the eastern parts of North America and is popular in different parts of the world. Chokeberries are rich sources of various phenolic acids, including anthocyanins, flavonols, flavanols, and proanthocyanidins, which are responsible for various health-promoting effects against noncommunicable diseases, including NAFLD [73]. 

Park et al. [74] revealed that the administration of chokeberry powder for eight weeks to HFD-fed mice attenuated the expression of genes for fatty acid synthase (FAS), SREBP, and acetyl-CoA carboxylase (ACC) in the liver. The elevated expressions of these genes contribute to the development of NAFLD. Moreover, supplementation with chokeberry powder decreased TG levels and fat droplet size in hepatocytes [74] (Table 1). Another study showed the mitigating effects of chokeberry on suppressing hepatic lipid accumulation by attenuating expressions of PPARγ2 factor and its downstream targets, including lipoprotein lipase (LPL) and adipocyte protein 2 (aP2), both in FFA-induced cell and HFD-induced animal models. Additionally, improvements in lipid levels and liver function, accompanied by reduced weight gain, have been observed with the use of chokeberry. The accumulation of lipids in FL83B cells was inhibited by 7% on giving a dose of 40 μg/mL and by 33.4% with a dose of 80 μg/mL. The study demonstrated for the first time the role of the PPARγ2-dependent molecular pathway in reducing hepatic lipid accumulation [75]. 

**Figure 5:**
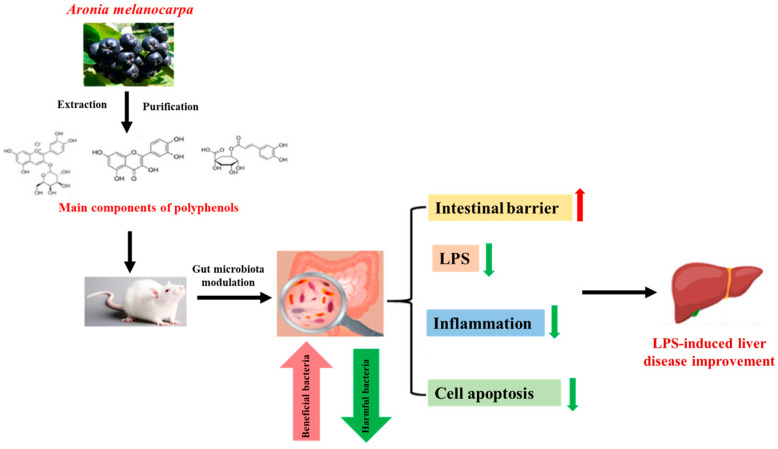
Effects of *Aronia melanocarpa* polyphenols (AMPs) on lipopolysaccharide (LPS)-induced liver diseases in rats. Supplementation of AMPs reduced LPS content, gene expressions of inflammatory markers, and apoptosis of liver cells, while promoting the intestinal barrier function and modulating the gut microbiota composition by increasing the level of beneficial bacteria. ↑: increase, ↓: decrease. Reproduced (with modifications) with kind permission from Ref. [78]. Copyright 2021 American Chemical Society.

Phenolic compounds have been shown to improve the intestinal barrier function and control dysbiosis of the gut microbiota [76,77]. Kong et al. [78] isolated polyphenols from chokeberry and demonstrated their hepatoprotective actions by modulating gut microbiota dysbiosis, enhancing intestinal barrier function, decreasing inflammatory marker expression, and reducing cell apoptosis in lipopolysaccharide-induced (LPS)-induced liver diseases in an animal model [78] (Figure 5) (Table 1). 

#### Clinical Study 

Boncheva et al. [79] documented the effects of daily supplementation of *A. melanocarpa* berry juice (200 mL, allocated into 3 servings, and administered 20 min before a meal) for 2 months in patients with NAFLD and applied a healthy lifestyle program more efficiently. Supplementation improves liver function, carbohydrate metabolism markers (including basal insulin, fasting blood glucose, and glycated hemoglobin (HbA1c)), and serum lipid parameters, including TC, HDL-C, LDL-C, TG, apolipoprotein A1, and apolipoprotein B [79].

### 5.6. Gooseberry 

Gooseberry (ambla, *Phyllanthus emblica* L.) is a member of the family Euphorbiaceae and is also known as *Emblica officinalis* or Indian gooseberry. This tree is native to India and grows in the tropical and subtropical regions of Southeast Asia, Pakistan, China, Uzbekistan, Sri Lanka, and Malaysia. The plant is blessed to have bioactive components, including vitamin C, tannins, lignans, alkaloids, flavonoids, ellagic acid, and mucic acid that provide beneficial effects such as antioxidant, hypolipidemic, hepatoprotective, antitumor, hypoglycemic, anti-inflammatory, and antimicrobial activities [80,81]. 

Oxidative stress is a major cause of NAFLD. A previous study revealed the antioxidant effects of the aqueous extracts of *Emblica offinalis* (dried berries) on human hepatoma cells (HepG2). The extracts possessed a high antioxidant capacity, which could be attributed to decreased levels of lipid hydroperoxides (LPHs) and ROS and increased glutathione levels, which are known to serve as non-enzymatic antioxidant defenses upon treatment. In addition, the treatment led to a higher expression of some common antioxidant enzymes, including glutathione peroxidase (GPx), superoxide dismutase (SOD), catalase (CAT), glutathione reductase (GR), and glutathione S-transferase (GST). This study showed the ability of gooseberries to modulate endogenous antioxidant defense systems [82] (Table 1). 

**Figure 6:**
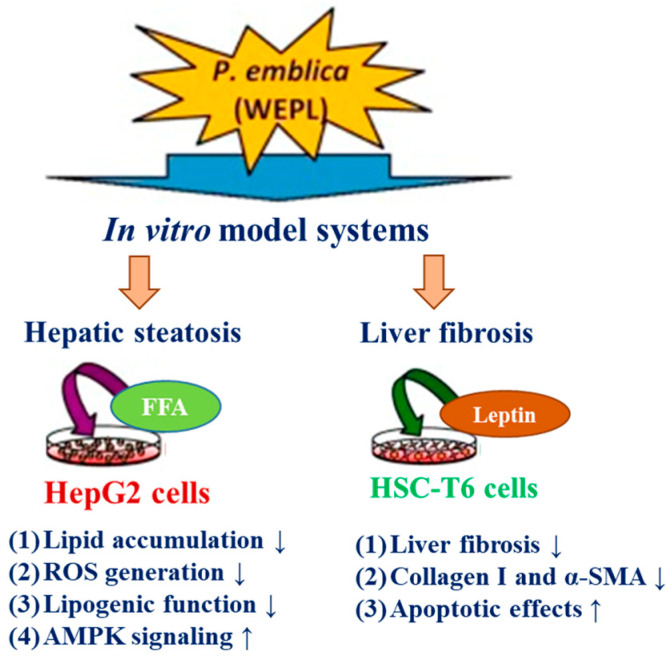
Inhibitory effects of the water extract from *P. emblica* fruits (WEPL) on in vitro model systems FFA-induced HepG2 cells and leptin-induced HSC-T6 cells. Supplementation of WEPL reduced lipid accumulation, ROS generation, and lipogenesis, and upregulated AMPK signaling in HepG2 cells while reduced fibrosis, expressions of collagen I and a SMA and increased apoptotic effects were witnessed in HSC-T6 cells. FFA: free fatty acid, ROS: reactive oxygen species, AMPK: 5′ AMP-activated protein kinase, α-SMA: alpha-smooth muscle actin. ↑: increase, ↓: decrease. Reproduced (with modifications) with kind permission from Ref. [83]. Copyright 2016 Elsevier.

Water extract from *P. emblica* fruits (WEPL) showed promising inhibitory effects on hepatic steatosis and liver fibrosis in an in vitro NAFLD model using HepG2 and rat hepatic stellate cells (HSC-T6) [83] (Figure 6). 

WEPL reduces cellular steatosis, ROS production, and lipid accumulation. PPARα is considered to play an essential physiological role in fatty acid metabolism and together with carnitine palmitoyl transferase-1 (CPT1) modulates the hepatic hypolipidemic mechanism by fatty acid β-oxidation [84]. Both markers were upregulated. However, proteins related to lipogenesis such as ACC, FAS, and phosphorylated AMPK, which work together with ACC, were downregulated in WEPL-treated FFA-induced HepG2 cells. Moreover, WEPL was found to inhibit liver fibrosis by decreasing collagen I, alpha-smooth muscle actin (α-SMA), and matrix metalloproteinase 2 expressions. In contrast, extract treatment induced the expression of apoptotic molecules such as Bax/Bcl-2 (Bcl-2 associated X, apoptosis regulator/B-cell lymphoma 2 protein), cleaved caspase-9, caspase-3, and poly (ADP-ribose) polymerase (PARP) in leptin-stimulated HSC-T6 cells. High-pressure liquid chromatography analysis of the WEPL revealed the presence of gallic acid and ellagic acid (EA) as the major compounds. The concentration-dependent treatments with EA show effects similar to those of the WEPL extract, such as decreasing lipid accumulation and ROS in FFA-induced HepG2 cells, decreasing collagen I and α-SMA, and increasing apoptotic molecules Bax/Bcl-2 and cleaved caspase-9, caspase-3, and PARP. These studies suggested that *P. emblica* has the potential to ameliorate NAFLD [83] (Table 1) (Figure 6). 

Huang et al. [85] demonstrated similar antioxidative properties of WEPE using an in vivo NAFLD model. HFD-induced SD rats when given WEPE administration showed lower levels of fat deposition, which resulted in lower body weights than HFD-treated SD rats. In addition, WEPE supplementation significantly downregulated serum AST, ALT, and LDL levels, whereas the expressions of the antioxidant enzymes CAT, GR, and GST were upregulated. Researchers found that the extract improved hepatic steatosis by increasing expressions of the lipid oxidation gene (PPARα) and lowering expressions of lipogenesis genes, namely liver X receptor-α (LXRα) and SREBP-1c in the liver [85] (Table 1).

Tung et al. [86] demonstrated the bioactive potential of WEPE in mice with MCD-induced NASH for up to eight weeks. The extract exhibited anti-inflammatory and antioxidant effects in WEPE-treated MCD-diet mice. The supplemented mice showed lower levels of AST, ALT, cholesterol, and common proinflammatory cytokines such as IL-1β and TNF-α, as compared to the MCD-diet group (Table 1). The enzymes involved in initiating oxidative stress, that is, cytochrome P450 2E1, and oxidative stress biomarkers, that is, thiobarbituric acid reactive substances, were downregulated compared to those in the MCD-diet group [86] (Table 1). These investigations show that *P. emblica* can slow the progression of NASH by reducing biochemical and histological markers of liver damage. However, further research, including clinical trials, is necessary to fully understand the therapeutic potential of *P. emblica*. 

### 5.7. Bilberry 

Bilberry (*Vaccinium myrtillus*) is a small edible berry that matures on small shrubs in central and northern European hilly areas. Bilberry contains around 15 species of anthocyanins, five species of anthocyanidins (cyanidin, delphinidin, petunidin, malvidin, and peonidin), and three different types of glycosides: glucoside, galactoside, and arabinoside [87]. The use of bilberries against NAFLD has been established both in vitro and in vivo. 

Haga et al. [88] demonstrated the effects of bilberry fruit extract (BE) on NASH in vitro and in vivo. The authors observed a reduction in steatosis by the dose-dependent suppression of cellular lipid accumulation in FFA-induced alpha mouse liver 12 (AML12) cells [88]. BE treatment improved cell survival and proliferation. However, BE was unable to reduce hydrogen peroxide-induced ROS, but could provide resistance against oxidative stress, as indicated by the significant activation of Nrf2. In animals, BE improved liver/body weight ratios, hepatic steatosis/TG content, liver fibrosis, and injuries in a dose-dependent manner. Fat metabolism-associated protein FAS was downregulated, and proteins involved in autophagy, such as run domain beclin-1-interacting and cysteine-rich domain-containing protein (suppressed later stage of autophagy) and p62/SQSTM1 (autophagy marker), showed a reciprocal trend, where BE induced p62/SQSTM1 expression and reduced rubicon expression in FFA-induced AML12 cells. In addition, LXR, SREBP-1c, FASN, and ACC-1 were upregulated upon FFA stimulation, which was considerably reversed by BE. In addition, Akt and STAT3 were phosphorylated and increased slightly, and manganese-dependent superoxide dismutase (MnSOD) and CAT were upregulated upon BE treatment, which contributed to cell survival and the antioxidation state. Another study revealed the role of bilberry anthocyanins (BA) in ameliorating NAFLD by playing an essential role in improving dyslipidemia and microbiome dysbiosis. BA supplementation showed antioxidant potential and remarkably reduced the ratio of Firmicutes/Bacteroidetes (F/B) and increased the relative abundance of *Akkermansia* and *Parabacteroide* [89]. These studies indicate the role of bilberry extracts in attenuating NALFD by improving the antioxidation state and modulating the gut microbiota. Further studies are required to demonstrate the effects of bilberry on signaling pathways and clinical markers to realize its full potential. 

### 5.8. Blackberry

Blackberry (*Rubus fruticosus* L.) is a commercially important perennial shrub cultivated globally. It is a member of the Rosaceae family. This plant has been documented to inhabit Asia, South and North America, Europe, and Oceania [90]. Its berries are rich in phenolic compounds, nutrients, and fibers, and have medicinal value [91]. Other names include European blackberries, bramble, wild blackberries, and shrubby blackberries. The beneficial properties of blackberries include antioxidant, anti-inflammatory, chemopreventive, anti-diabetic, and anti-obesity activities and protection against neurodegeneration [92,93]. 

The effects of blackberry plant extract were assessed using different NAFLD models. Blackberry leaf ethanol extract was more effective than blackberry fruit extract in reducing TG accumulation in HepG2 cells. Additionally, TG accumulation was decreased by a mixture of blackberry leaf and fruit extracts at a lower dose than by the individual extracts. Notably, blackberry fruits enhanced the overall SCFA content and butyrate and propionate production, whereas blackberry leaves boosted propionate production during fecal incubation. Blackberry fruit increased the contents of Bifidobacteriales and Lactobacillales and decreased the content of Clostridiales, while blackberry leaves increased the content of Bacteroidales and decreased that of Enterobacteriales [94] (Table 1). Using an in vivo model, blackberry leaves and their mixture (leaf and fruit) reduced HFD-induced liver damage by protecting against liver injury induced by oxidation and inflammation. The mixture increased the abundance of *Lactobacillus* and *Akkermansia*, propionic acid, and butyric acid, resulting in the amelioration of systemic inflammation and oxidative stress and the reduction in NAFLD phenotypes [95]. These studies suggest that future research should assess the potential of blackberry fruit and leaf extracts against liver diseases and well-designed clinical trials should be conducted. 

### 5.9. Açai

Açai (*Euterpe oleracea* Mart.) berry has gained attention because of its high polyphenol content, which mainly comprises anthocyanins, proanthocyanidins, and other flavonoids. The berry is obtained from a palm tree in the Amazon region and is commonly known as the Brazilian berry. The health-promoting functions of this wonder berry have been attributed to the presence of anthocyanins and flavonoids [96]. 

Considering the high anthocyanin content, many studies have utilized various extracts of açai berry against NAFLD. One study produced an aqueous extract and determined 118.13 mg gallic acid equivalents (GAE)/100 g and 9.23 mg/100 g as the total phenolic and flavonoid contents, respectively. In an HFD-induced steatosis mouse model, the aqueous extract improved insulin sensitivity, increased adiponectin levels, and increased PPAR-mediated fatty acid oxidation, demonstrating its liver-protective properties against steatosis [97] (Table 1). Increased adiponectin levels are considered to be a substantial link with NAFLD prevention. However, the study did not evaluate the possible effects on antioxidant enzymes. Considering this limitation, Pereira et al. [98] worked on evaluating the protecting effects of açai berry on paraoxonase (PON) isoforms and PON1 activity. PON is a calcium-dependent esterase that is strongly linked to apolipoprotein A-I and HDL, and confers antioxidant characteristics by reducing the buildup of lipid peroxidation products [99]. The filtered açai pulp contained total polyphenol (458.6 mg GAE/100 g) and monomeric anthocyanin (13.59 mg/100 g). The pulp exhibited dose-dependent 2, 2-diphenyl-1-picrylhydrazyl radical scavenging activity. The authors discovered that the açai pulp has protective effects against oxidative stress by upregulating PON1 and preventing LDL oxidation, relieving hepatic steatosis [98] (Table 1). 

Another study demonstrated the attenuating effects of açai treatment on the degree of steatosis, inflammation, and oxidative stress by increasing the activity of catalase and the reduced glutathione/oxidized glutathione ratio in the fructose-rich diet-induced rat model [100]. Similar results were reported for an aqueous extract of açai in vitro and in vivo [101]. Of note, Barbosa and coworkers demonstrated that the maternal diet with açaí supplementation can attenuate lipid buildup in the liver, reducing the advancement of NAFLD in dams and protecting the offspring [102]. In a follow-up study, researchers examined antioxidant defense mechanisms and indicators of oxidative stress and discovered that after receiving açaí treatment, oxidative stress biomarkers were found to be reduced in the livers of the dams, whereas açaí treatment raised Gpx1, Gpx4, and SOD1 expression in the offspring [103] (Table 1). Future research should assess the effects of açai berry through RCT-designed clinical studies. 

### 5.10. Blackcurrant

Blackcurrant (*Ribes nigrum* L.) contains high amounts of phenolic compounds such as anthocyanins. Blackcurrant originated in Europe and northern Asia and has been popular in the United States since the early 2000s [104]. Given the high anthocyanin content of blackcurrant berries, they exhibit remarkable antioxidant, hypocholesterolemic, and anti-inflammatory effects [105]. 

Blackcurrant berries, as shown in prior studies, possess anti-inflammatory and hepatic steatosis-preventing properties [106,107]. In a follow-up study, Lee et al. evaluated the effects of blackcurrant extract (BCE) in a mouse model of obesity-induced nonalcoholic steatohepatitis. According to previous studies, feeding mice with an obesity-induced diet together with 6% whole blackcurrant extract for 24 weeks reduced the infiltration of proinflammatory M1 macrophages into the liver and decreased the expression of fibrogenic genes and fibrosis in the liver. Additionally, BCE drastically decreased hepatic micro RNA (miR)-122-5p and miR-192-5p NAFLD markers. Moreover, the LPS-stimulated production of proinflammatory genes in splenocytes isolated from the HFD and blackcurrant groups was considerably reduced [108] (Table 1). However, further studies are required to assess the full potential of these berries against NAFLD. 

Menopausal women with low estrogen secretion typically gain weight and develop steatosis because of poor lipid metabolism. Nanashima et al. [109] examined the results of a three-month dietary experiment using ovariectomized (OVX) rats to assess the effects of BCE (3%). Proinflammatory cytokine gene expression was lower in the treated OVX rats than in the control group. Rats fed with BCE had considerably lower NAFLD activity scores than those in the OVX control group (Table 1). To the best of our knowledge, this is the first study to demonstrate that BCE consumption can successfully prevent abnormalities in lipid metabolism and liver steatosis in a rat model of menopause. However, further studies are required to validate its clinical efficacy [109].

**Table 1:** Effects of different berry plant parts on non-alcoholic fatty liver disease.

Ref.	Extract/Disease	Study Model	Dose	Biological Response
[43]	Aqueous and ethanolic extract of *Lycium barbarum* (LBAE and LBEE)/oxidative stress	Male Wistar rats	Normal control group High-fat diet (HFD) group HFD + simvastatin (20 mg/kg BW) group HFD + LBAE (50 mg/kg BW) group HFD + LBAE (100 mg/kg BW) group HFD + LBEE (50 mg/kg BW) group HFD + LBEE (100 mg/kg BW) group, 8 weeks	No significant difference in body weight. ↓severity of hepatic lesions, ↓TC, ↓TG, ↓LDL-C, ↑HDL-C, ↓AST, ↓ALP, ↓ALT, ↓Liver MDA, ↑liver GSH, ↑SOD, ↑CAT, ↑GSH-Px, and ↑TAOC. LBEE groups showed better results than LBAE groups.
[44]	*Lycium barbarum* polysaccharides (LBP)/NASH	Female SD rats	Control group HFD group Vehicle group (LBP 1 mg/kg, one time/day) HFD + LBP (1 mg/kg), 8 weeks	No significant difference in body weight. ↓fat deposition, inflammation, and collagen formation within the liver. ↓SREBP-1c, ↓PPARγ2, ↑ATGL, ↑adiponectin, ↓pSMAD2, ↓pSMAD4, ↑CAT, ↑GPx, ↓CYP2E1, ↓TNF-α, ↓IL-1β, ↓COX-2, ↓iNOS, ↓p-ERK, ↓p-JNK, and ↓p-c-Jun.
[45]	*Lycium barbarum* polysaccharides (LBP)/NASH	Female SD rats	Control group NASH group (HFD for 12 weeks) Vehicle group (LBP 1 mg/kg, one time/day, whole experiment period) Vehicle therapeutic group (LBP 1 mg/kg, one time/day, 9th to 12th week) HFD, + LBP (1 mg/kg), 12 weeks HFD+ therapeutic LBP (1 mg/kg), 9th to 12th week	Reduced body and liver weights of NASH mice. Improved insulin resistance, normalized blood glucose level, improved hepatic histopathology, ↓pSMAD2, ↓pSMAD4. ↓SREBP-1c, ↓PPARγ2, ↑ATGL, ↑adiponectin. ↑CAT, ↑GPx, ↓MDA, ↓TNF-α, ↓IL-1β, ↓COX-2, ↓MCP-1, ↓cleaved caspase-3, ↓Bax-1, ↑Bcl-2, ↓NF-kB, ↓p38 MAPK, ↓p-JNK, and ↓p-ERK1/2.
		BRL-3A cells	Control group Steatosis group (Sodium palmitate acid (0.35 mM PA), 24 h Vehicle-LBP group (LBP only); Steatosis 1 LBP group (PA + LBPs, 24 h) Vehicle-arabinose group (l-arabinose (3 mM) only) Steatosis 1 arabinose group (PA + l-arabinose) Vehicle-carotene group (β-carotene (2 mM) only) Steatosis 1 carotene group (PA and β-carotene (2 mM)), 24 h	Restored cell viability, ameliorated insulin and glucose metabolism, ↓fat accumulation, ↑CAT, ↓TNF-α, And ↓apoptosis. LBP showed stronger protection than β-carotene or 1-arabinose.
[46]	*Lycium barbarum* polysaccharides (LBP)/NAFLD	Male C57BL/6 mice	Control group HFD group HFD+ LBP (100 mg/kg) group HFD+ LBP (200 mg/kg) group, 12 weeks	↓TG levels in the serum and liver, ↑SIRT1, p-AMPK, ACC, and ATGL expressions, ↓FAS, blocked hepatic lipogenesis, and activation of SIRT1/AMPK pathway.
		HepG2 cells	25 μM palmitic acid (PA), 12 h LBP (30, 100, 300, 600, 900 μg/mL), 24 h	↑SIRT1 deacetylase activity, ↑NAD+/NADH, and ↑ NAD+ levels (dose-dependent). ↑LKB1 deacetylation, ↑pAMPK, ↑pACC, ↓FAS, and ↑ATGL.
[47]	*Lycium barbarum* polysaccharide (LBP) and olive oil/Liver fibrosis	Male SD rats	Normal group Soybean oil group (CCl_4_, 1.0 mL/kg BW + soybean oil 4%) Olive oil group (CCl_4_ + olive oil 4%) Mixed oil group (CCl_4_ + 2% olive oil + soybean oil 2%) LBP (50 mg LBP/kg BW/day) and soyabean oil group (4% SO + LBP (S + L) LBP and olive oil group (4% olive oil + LBP (O + L) LBP and mixed oil group (Mixed oil + LBP (M+L), 9 weeks	LBP-inhibited caspase-9/3 activities, ↓TNF-α in liver, ↑IL-10, IL-10/TNF-α ratios, ↓TGF-β1, and TIMP-1 levels. LBP + olive oil = ↓liver apoptotic and inflammatory markers and attenuated hepatic TGF-β1 levels
[48]	*Lycium barbarum* polysaccharides (LBP)/NASH	C57BL/6N mice	Control group Vehicle LBP group (LBP 1 mg/kg once a day) Methionine-choline deficient (MCD) group (MCD diet, 6 weeks) MCD + LBP group: MCD treatments first three weeks) + 1 mg/kg LBP from 4th to 6th week, once a day	Improved hepatic injury and fibrosis. ↓MDA level, restored CAT, GPx1, ↓Txnip ↓TNF-α, ↓NF-κB-p50, ↑IκB-α, ↓caspase-3/7, ↓Bax-1, ↓cytochrome c, ↓CYP2E1, ↑Bcl-2, ↓serum levels of IL-18/IL-1β, ↓cleaved caspase-1, ↓ASC, ↓NLRP3, and ↓NLRP6 in the liver.
[49]	*Lycium barbarum* polysaccharides (LBP)/NAFLD	Male SD rats	Control group HFD group HFD + LBP group (50 mg/kg) HFD + medium intensity aerobic exercise (AE) group HFD + LBP aerobic exercise group, 8 weeks	Inhibiting inflammation and regulating host gut microbiota. ↑Bacteroidetes, ↑short-chain fatty acids, ↓Proteobacteria, and ↓Firmicutes/Bacteroidetes. Restored tight junction protein expressions. Observed additional benefits for LBP + AE than LBP or AE.
[69]	Blueberry juice (BBJ) + *Bifidobacterium* (*B.*) *lactis*, *Lactobacillus bulgaricus, Streptococcus thermophilus*/NASH	Male SD rats	Control group Model rat group (saline solution (50 µL/kg) (I.P.) + liquid placebo (20 mL/kg) daily) BBJ group (saline + BBJ (10 mL/kg) + liquid placebo (10 mL/kg) daily) BBJ and PB group (Saline+ BBJ + P (10 mL/kg) daily) PPAR-α inhibitor group (PPAR-α (50 µL/kg) in saline BBJ and PPAR-α inhibitor group (PPAR-α + BBJ + liquid placebo daily) BBJ, P, and PPAR-α inhibitor group (PPAR-α in saline + BBJ+ P daily)	↓size of lipid droplets, ↑SOD, ↑GSH, ↑HDL-C, ↓AST, ↓ALT, ↓LDL-C, ↓TG, ↓MDA, ↑PPAR-α, ↓SREBP-1c, ↓PNPLA-3,↓IL-6, ↓TNF-α, ↓caspase-3, and ↓Bcl-2. Increased protecting effects of BBJ + PB.
[70]	Blueberry juice + *B. infantis*, *B. animalis* and *Lactobacillus acidophilus*/NAFLD	SD rats	Control group (CG) HFD group (MG) HFD + blueberry juice (1.5 mL per 100 g weight) once a day, 10 days (BG) HFD + blueberry juice + probiotics 250 mg/100 mL and 20 mL/100 g) once a day, 10 days (BPG)	Reversed hepatic mitochondrial damage, mitochondrial swelling, and hepatic necrosis. ↓MDA, ↓ROS, ↑GSH, ↑SOD, ↑PGC-1α. Antioxidative effect: Juice + probiotics was stronger than blueberry.
[71]	Blueberry *B. infantis*, *B. animalis* and *Lactobacillus acidophilus*/NAFLD	Male SD rats	Normal control HFD+ lentivirus carrying siRNA NC HFD + SiRNA NC + blueberry and probiotics (BP) HFD + Si RNA HFD + SiRNA + BP Lentivirus carrying siRNA (1 × 10^8^) at a time on alternate days, 12 weeks + BP (1.5 mL/100 g weight, once a day with probiotics at 10^8^ CFU/mL), 8 weeks	↓lipid deposition, ↓TG, ↑IL-22, ↑JAK1, ↑STAT3, ↓Bax ↓ALT, and ↓AST.
		L-02	Normal group FFA + siRNA group (negative control) FFA + IL-22 siRNA group FFA + siRNA negative control + BPS group FFA + IL-22 siRNA + BPS group, 24 h, FFA (oleic acid (OA):palmitic acid (PA) = 2:1, 1 mM/L), 24 h	↓lipid deposition, ↑IL-22, and ↓TG
[72]	Malvidin-3-*O*-glucoside (M3G) and M3Ga from blueberry/NAFLD	L-02	FFA (200 μM) + M3G (10, 100, 200, and 400 μM), 24 h	Inhibitory effect on ROS by M3G, M3Ga, ↑GSH compared to FFA treated group. M3G: ↑lysosomal biogenesis (dose-dependent). ↑LAMP1, TFEG (↑ Nuclear, ↓ Cytoplasmic), ↑HO-1, ↑NQO1, ↑γ-GCL, ↑SOD, ↑GPx, and ↑CAT.
[74]	Chokeberry powder/NAFLD	Male C57BL/6J mice	Normal diet group High cholesterol and HFD group (HF) HF + powder (0.5%) HF + powder (1%), 8 weeks	↓fat droplet size in the liver, ↓TG, ↓SREBP, ↓ACC, and ↓FAS. Not affected dose-dependently.
[75]	Ethanol extract of *Aronia melanocarpa*/NAFLD	Male C57BL/6N mice	Normal diet group HFD group HFD + extract (50 mg/kg daily), 12 weeks	↓body weight, ↓liver weight, ↓TG, ↓FAS, ↓ALT, ↓AST, ↓leptin, ↑SOD, ↑TEAC, ↓PPARγ2, ↓aP2, and ↓LPL.
		FL83B cells	FFA + extract (40, 80 μg/mL), 24 h	↓intracellular lipid droplets, ↓PPARγ2, ↓aP2, and ↓LPL.
[78]	*Aronia melanocarpa* polyphenols (AMP)/Liver disease	Male SD rats	Control group Model group (LPS, 200 μg/kg) Low-dose group: LPS (200 μg/kg) + AMP (50 mg/kg BW) Medium-dose group: LPS (200 μg/kg) + AMP (100 mg/kg BW) High-dose group: LPS (200 μg/kg) + AMP (200 mg/kg BW), 4 weeks	Dose-dependent effect: ↓IL-6, ↓IL-1β, ↓TNF-α. Dose-dependent effect: ↓cleaved caspase-3, ↓Bax, ↓cleaved PARP, ↑Bcl-2. Dose-dependent effect: ↓TLR4, ↓MyD88, ↓p-STAT3, ↓ROS, and ↑GSH. ↑claudin-1, no significant difference for Zonula Occludens-1 and occludin. ↑*Lactobacillus*, ↑*Enterobacteriaceae*. ↓*Lachnospiraceae*, ↓*Phascolarctobacterium*, and ↓*Clostridiales.*
[82]	*Emblica officinalis*/NAFLD	HepG2 cells	Aqueous extract of *E. officinalis* (5, 10, 20, 50, and 100 μg/mL), 4, 8, 12, 16, 20, and 24 h	↑cell death, ↑LDH, ↓ROS, ↑antioxidant capacity, ↑glutathione, ↑SOD, ↑CAT, ↑GPx, ↑GR, and ↑GST.
[83]	Water extract of *Phyllanthus emblica* L. fruits (WEPL)/NAFLD	HepG2 cells	FFA (OA:PA, 2:11 mM) + WEPL (50, 100, and 200 μg/mL), 48 h	↓cellular steatosis, ↓ROS, ↓lipid accumulation, ↑PPARα, ↑CPT-1, ↓ACC, ↓FAS, and ↑pAMPK.
		HSC-T6 cells	Leptin (100 ng/mL) + WEPL (50, 100 and 200 μg/mL), 24 h	↓collagen I, ↓α-SMA, ↓MMP-2 activation, ↑cytotoxicity, ↑caspase-9, ↑caspase-3, and ↑Bax/Bcl-2.
		HepG2 cells	FFA (1 mM, 12 h) + Ellagic acid (EA, 2.5, 5 and 10 μM), 48 h	↓lipid accumulation and ↓ROS.
		HSC-T6 cells	Leptin (100 ng/mL) + EA (2.5, 5 and 10 μM), 24 h	↓collagen I, ↓α-SMA, ↑Bax/Bcl-2, ↑caspase-9, ↑caspase-3, and ↑PARP.
[85]	Water extract of *P. emblica* fruit (WEPE)/NAFLD	Male SD rats	Control group HFD group HFD + gallic acid (100 mg/kg BW) HFD + WEPE (L: 125 mg/kg BW) HFD + WEPE (M: 250 mg/kg BW) HFD + WEPE (H: 500 mg/kg BW), 20 weeks	↓body weight and ↓food efficiency ratio. ↓peritoneal and epididymal fat pads (significant reduction with medium and high doses). ↓AST (WEPE extracts). ↓ALT, ↓LDL, and ↑glutathione reductase (H: WEPE-significant). ↑CAT (all doses), ↑GST and ↑adiponectin (all WEPE doses and gallic acid). ↑PPARα (M-WEPE), ↓LXRα (H-WEPE), ↓SREBP-1c (all WEPE groups).
[86]	Water extract of *P. emblica* fruit (WEPE)/NASH	C57BL/6 mice	Control group MCD group MCD + gallic acid group MCD diet + low-dose (125 mg/kg BW) group MCD diet + medium-dose (250 mg/kg BW) group MCD + high dose (500 mg/kg BW), 4 or 8 weeks	↓AST, ↓ALT, ↑serum cholesterol, ↑serum TG, ↑CAT, ↑GPX, ↑SOD, ↓TBARS, ↓hepatic CYP2E1, ↓IL-1β, and ↓TNF-α.
[88]	Ethanolic extracts of bilberry (BE)/NASH	Alpha mouse liver 12 cells	T090 (1 μM, LXRα agonist) or Fatty acids (100 μM), BE (5 μg to 10 μg), 3–5 days.	↓LXR, ↑cell survival/proliferation, and ↓steatosis (dose-dependently).
		Leptin receptor-deficient (BKS.Cg-+ Leprdb/+ Leprdb/Jcl; db/db) mice	Normal control diet group High-fat and high-cholesterol diet (HF-HC) group HF-HC diet + BE (5%) HF-HC diet + BE (10%), 8 weeks	↓lipid accumulation, ↓oxidative stress, and ↓Nrf2. Improved liver/body weight ratios, hepatic steatosis/TG contents, liver fibrosis ↓FAS, ↑rubicon, ↑p62/SQSTM1, ↓SREBP-1c, ↓FASN, ↓ACC-1, ↑Akt, ↑STAT3, ↑MnSOD, and ↑CAT.
[89]	Bilberry anthocyanins (BA)/NAFLD	C57BL/6N mice	Normal diet Normal diet + BA (2%) Western diet Western Diet + BA (2%), 18 weeks	↓body weight, ↓liver weight, ↓epididymis fat weight, and ↓lipid accumulation. ↓AST ↓ALT ↓MCP-1. ↓LDL-c, ↑HDL-c, and ↓TG. ↓insulin, ↓insulin resistance ↑Nrf2 ↑SOD2 ↓Keap-1, ↓TBARS, ↓α-SMA, ↑caecal weight, ↓Firmicutes/Bacteroidetes, ↑*Bacteroides* acidifaciens, ↑*Parabacteroides distasonis*, ↑*Akkermansia muciniphila*, ↓*Prevotella*
[94]	Blackberry leaf (BL) and fruit (BF) extracts/NAFLD	HepG2	Palmitate (0.5 mM), 24 h + pretreatment with BF and BL or mixture (BF and BL = 1:2) (30 or 90 μg/mL), 1 h	Improved cell viability and ↓TG. ↓ACC, ↓FAS, ↓SREBP-1c, ↑CPT-1, ↑SOD, ↑GSH-Px, ↓MDA, ↓TNF-α, and ↓IL-1β. ↑SCFA, butyric acid, and propionic acid. Improved gut microbiota composition. BF: ↑Bifidobacteriales, ↑Lactobacillales, ↓Clostridiales BL: ↑Bacteroidales, ↓Enterobacteriales
[95]	Blackberry leaf and fruit extracts/NASH	SD rats	HFD + 50% ethanol leaf extract (450 mg/kg BW), 50% ethanol fruit extract (450 mg/kg BW), mixture group (2:1, 150 mg/kg BW) Dextrin (NAFLD control, 450 mg/kg BW; control), Positive control, milk thistle extracts (150 mg/kg BW) Normal control	Protection against liver enlargement. ↑CPT-1, ↓ACC, ↓FAS, ↓SREBP-1c. ↑SOD, ↑GSH-Px, and ↑GSH. ↓TNF-α, ↓IL-1β, ↓ALT, ↓AST, ↓TC, ↓LDL-C, and ↓HOMA-IR. ↑*Akkermansia* in all treatment controls than NAFLD control and ↑*Lactobacillus* higher in the mixture group than other groups.
[97]	Açai aqueous extract (AAE)/NAFLD	Male Swiss mice	Control group HFD group HFD + AAE (3 g/kg) once daily, 12 weeks	Improved insulin resistance and ↑adiponectin. ↓TG and ↓lipid droplets. ↑AdipoR2, ↑PPAR-α, ↑CPT-1α, ↓SREBP-1c, ↓FAS, and ↓ACC1.
[98]	Açai pulp/Hepatic steatosis	Rats Fischer 344 (F344)	Control group CA group (açai pulp, filtered (2 g/day) HF group (HFD diet) HFA (HFD + açai pulp, filtered (2 g/day)), 6 weeks	↓ALT, ↓total liver fat, ↓TG, and ↓ox-LDL levels. ↑PON1, ↑PON3, ↑APOA-1, and ↑arylesterase activity of PON1.
[100]	Açai/NAFLD	Male Fischer rats	Control group Fructose-rich diet (60%) + lyophilized açai (2%), 10 weeks	↓ALT, ↓AST, and ↓degree of steatosis.
[101]	Aqueous açai extract/NAFLD	Male Swiss mice	Control group HFD group Standard diet + extract (3 g/kg) single daily dose HFD + extract (3 g/kg) single daily dose, 6 weeks	↓TNF-α, ↓liver weight, and liver fat percentage. ↓MDA/TBARS. Modulated GR, SOD, and CAT.
		HepG2 cells	TBHP + extract (50 and 100 mg/mL), 24 h	Inhibited ROS production.
[102]	Açaí pulp/steatosis	Female Fisher rats, male offspring after birth (P1) and weaning (P21)	Control diet HFD diet Control diet + açaí pulp (2%) HFD + açaí pulp (2%)	↓liver weight, ↓fat, and ↓cholesterol. ↑Srebpf1, ↑Sirt1, and ↑Fasn. Offspring (HF+ pulp diet) ↓liver weight and serum cholesterol only in P21 (weaning) not in P1 (after birth).
[103]	Açaí pulp	Female Fisher rats dams and offspring (P21)	HFD + açaí pulp (2%), 21 days	↑Gpx1, ↑Gpx4, and ↑SOD1 in offsprings.
[108]	Whole blackcurrant powder/obesity-induced NASH	Male C57BL/6J mice	Low-fat diet group Low-fat diet + blackcurrant powder (6%) HFHD group HFHS + blackcurrant powder (6%), 24 weeks	↓plasma ALT, ↓liver weight, ↓TG, and ↓ACC. ↓macrophage markers: F4/80, Cd11c, and CCL2. ↓fibrogenic genes: *Col1a1*, *Col6a1*, and *Col6a3*. ↓TGFβ1. ↓Hepatic miR-122-5p and miR-192-5p.
		Ex-vivo isolated splenocytes	LPS (500 ng/mL), 3 h	↓IL-1β and ↓TNF-α. ↓IL-6 (non-significant).
[109]	Blackcurrant extract (BCE)/Dyslipidemia and steatosis	SD OVX female rats	AIN-93M diet group AIN-93M diet group + BCE (3%), 3 months	↓Body weight and ↓visceral fat weight. ↓serum TG, ↓TC, and ↓LDL-C. ↓adipocytes diameter. ↓TNF-α, ↓IL-6, and IL-1β. ↓adipocytokines.
[110]	Acerola polysaccharides (ACP)/NAFLD	Male C57BL/6 mice	Normal diet group Normal diet + ACP (800 mg/kg/day) HFD group HFD + ACP (200 mg/kg/day) HFD + ACP (400 mg/kg/day) HFD + ACP (800 mg/kg/day), 9 weeks	↓body and liver weights. ↓glucose level (middle and high dose). Serum: ↓insulin level, ↓TG, ↓TC, ↓LDL-C (high dose), and ↓serum ALT (high dose). Hepatic ↓TC, ↓TG, and ↓NEFA (high dose). ↓SREBP1c, ↓ACC, ↓FAS, and ↓SCD-1. ↓TNF-α, ↓IL-6, and ↓IL-1β. ↑GSH/GSSG, ↑SOD, and ↑CAT. ↑Nrf2, ↑HO-1, and ↑NQO-1. ↑complex V. ↓UCP2. ↑PGC-1α.
[111]	Cranberry extract (CBE)/NAFLD	Male C57BL/6J mice	HFD die group HFD + CBE (0.8%), 21 weeks	↓FAA and ↓IL-1β. ↓ALT. ↓Total lipid droplet area in the liver. ↓CCL2. ↓TNF-α, ↓COX-2, ↓IL-1β, and ↓UCP2. ↓NOS2 and ↓CCL2 (non-significant). ↓*TLR4* and ↓*NF-kB*. ↓*Ccr2* and ↓*Ccl3*. ↓*NLRP3*, ↓*Txnip*, ↓*Pparα*, and ↓*Casp1*. ↓caspase 1 (non-significant).
[112]	Cranberry nutraceuticals/NAFLD	Male albino Wistar rats	Normal diet group HFCD group HFCD + cranberry (50 mg/kg/day) 3 times per week, 8 weeks HFCD + cranberry (100 mg/kg/day), 3 times per week, 8 weeks Normal cho diet + cranberry (100 mg/kg/day), 3 times per week, 8 weeks	↓AST, ↓ALT, and ↓TG. ↓MDA, ↑GSH, ↑CAT, and ↑SOD. ↓TNF-α, ↓IL-6, and ↓NF-kB. ↑ADP. ↓TGF-β, ↓α-SMA, and ↓hydroxyproline.
[113]	Cranberry powder/NAFLD	Male C57BL/6 mice	Normal diet group HFD group HFD + cranberry powder (1%) group HFD + cranberry powder (5%) group, 8 weeks	↓IL-6, ↓serum TG, ↓SREBP-1, and ↓lipid droplets. ↓PPARγ and ↓MCP-1.
[114]	*G. mangostana* pericarp ethanolic Extract/liver cirrhosis	Male/Female SD rats	Normal control TAA + Tween 20 (10%) TAA + silymarin TAA (200 mg/kg), 8 weeks (three times weekly) + extract (250 mg/kg), 2 months TAA + extract (500 mg/kg), 2 months	↓PCNA, ↓α-SMA, and ↓TGF-β1. ↑SOD, ↑CAT, and ↓MDA. Restored ALP, ALT, and AST.
[115]	*G. mangostana* pericarp extract/NAFLD/NASH	Male Balb/c mice	Normal diet group HFD group HFD + extract (50 mg/kg/day), 16 weeks	↓body weight and liver weight. ↓ALT and ↓AST. ↓CD68 positive macrophages. ↓α-SMA expression. ↓FFA, ↓TG, ↓TC, ↓LDL-C, and ↑HDL-C.
[116]	Lingonberry powder (air-dried)/NAFLD	Male C57BL/6N mice	Low-fat diet group HFD group HFD + LGB (20%) *w*/*w* group, 11 weeks	Prevented weight gain and liver weight gain. ↓serum ALT. ↓Lepr. ↓Saa1 and ↓Saa2. ↑Cyp3a11, ↑Cyp2c55, ↑Cyp2c29, ↑Cyp3a59, and ↓Cyp46a1. ↑Hsd17b6 and ↑Igfbp2. ↓Plin4 and Mogat1-lipid metabolism. ↓Lcn2, ↓Cxcl14, and ↓S100a10–inflammatory/immune response or cell migration. ↓Cdkn1a, ↓Tubb6, ↓Tubb2a, regulation of cell cycle.
[117]	Lingonberry/NAFLD	C57BL/6J male	Control group HFD group HFD + berry (5% *w*/*w*), 12 weeks	↓ALT and ↓AST. ↓plasma lipid levels. ↓TG and cholesterol. ↑lipid droplets compared. ↓ACC-1, ↓SREBP-1c. ↑pAMPK. ↓MDA. Restored GSH. ↑GSH/GSSG. ↑Gclc and ↑Gclm. Restored Nrf2. ↓IL-6, ↓MCP-1, and ↓TNF-α.
[118]	Lingonberry extract (LBE)/NAFLD	C57BL/6J mice	Control group HFD group HFD + LBE (5% *w*/*w*), 12 weeks	↓Notch1, ↓NICD1, and ↓HES. ↓TG and ↓TC in liver. ↓SREBP-1c and ↓ACC1. ↑ACOX1 and ↑CPTIα. ↓CD36, ↓DGAT1, and ↓DGAT2.
		HepG2	Pretreated with LBE (1:2000, 1:1000 and 1:500) + C3Glu (0.1 and 1.0 M) and γ-secretase inhibitor, 30 min, + PA, 24 or 48 h	↓Notch1. ↓SREBP-1c, ↓ACC1, and ↓HES. ↓TG. ↓lipid droplets.
[119]	Maoberry extracts/NAFLD	Male SD rats	HFD group HFD, 4 weeks + extract (0.38 (ML), 0.76 (MM), 1.52 g/kg BW (MH)) HFD group + statin (10 mg/kg), 12 weeks	↓oxidative stress (MH significant), ↓TNF-α, ↓IL-6, ↓ACC ↓GPAT-1, and ↓SREBP-1c. ↓TG (MH group). ↓AST.
[120]	Lyophilized maqui/NAFLD	C57BL/6J male mice	HFD group HFD + extract (4 mg/day), 16 weeks	↓hepatic TG. ↓*Cpt1a*, ↓*Ehhdah*, and ↓*Pparα*. ↓Fasting glucose levels and ↓G6Pase. ↑*smile* and ↓*pgc1α*.
[121]	Raspberry ketone/NASH	SD rats	Normal control group HFD group HFD + raspberry ketone low-dose (0.5%) group HFD+ raspberry ketone middle dose (1%) group HFD+ raspberry ketone high-dose (2%) group, 8 weeks	↓AST, ↓ALT, ↓ALP, ↓Glucose, ↓insulin, ↓IR, ↓Insulin-Sensitive Index, ↓leptin, ↓FFA, ↓TNF-α, ↓MDA (high dose effective), ↑SOD, ↑adiponectin ↑PPAR-α, ↑LDLR, and ↓hs-CRP. All doses were effective.
[122]	Raspberry ketone (commercial)/NAFLD	Male Wistar rats	NC group = normal control diet (NCD) FLC group: HFFD diet FLN group: HFFD (7 weeks) shifted to NCD (8 weeks) FLCR group: HFFD (7 weeks) shifted to calorie-restricted diet (8 weeks) FLRK group: Rats fed HFFD (7 weeks), shifted to normal chew diet + RK (55 mg/kg/day, orally), 8 weeks	↓body weight, ↓liver tissue, ↓TC, ↓TAG, ↓LDL-C, ↑HDL-C, ↓ALT, ↓AST, ↓SREBP-1c, ↑lipid oxidation, ↓FAS, ↑PPAR-α, ↑CPT-1, and ↑p-AMPK.
[123]	Raspberry ketone (RK)	Male C57BL/6J mice	TAA (100 mg/kg, 3 times/week), 2nd to 5th week (200 mg/kg, 2 times/week) + RK (10, 20, and 40 mg/kg), and positive group TAA + curcumin (20 mg/kg), and individual group, RK (40 mg/kg)	↓AST, ↓ALT, ↓hydroxy proline, ↓α-SMA, ↓collagen I, ↓TIMP-1, ↓TIMP-1/MMP13, ↓Cleaved caspase-1, ↓IL-6, ↓IL-1β, ↓MPO, ↓IL-18, and ↓TNF-α. Inhibited EMT ↑E-cadherin, ↑β-catenin, ↑ZO-1, ↓N-cadherin, and ↓vimentin. ↓PPARγ, ↑FXR, ↑PGC-1α, and ↑SHP. Activated FXR-PGC-1α signaling
		HSC-T6, LX-2, and THP-1	HSC-T6 + pretreated with TGF-β (10 ng/mL), 2 h, and THP-1 + LPS pretreatment (10 ng/mL), 6 h + RK (6.25, 12.5, 25, and 50 μM), 24 h	RK (50 μM) ↓α-SMA, ↓collagen I, ↓TIMP-1, and ↓TIMP-1/MMP13. ↓Cleaved caspase-1, ↓IL-1β, and ↓IL-18. Inhibited EMT ↑E-cadherin, ↑β-catenin, ↑ZO-1, ↓N-cadherin, and ↓vimentin. ↓PPARγ, ↑FXR, ↑PGC-1α, and ↑SHP.
[124]	Raspberry extract (RE)/NAFLD	Male Wistar rats	HFLF + RE (0.64%) (HP diet). HFLF + RE + Fructooligosaccharides (FOSs, 3%) (HPF diet) HFLF + RE + pectin (3%) (HPP), 12 weeks	↓PPARα, ↓ANGPTL4, ↓PPARλ, ↓SREBP1c, ↓AST, ↓TG, and ↓IL-6 (mainly HPF>HP>HPP). RE+ FOSs = more favorable effect.
[125]	Raspberry extract + FOSs/Advanced NAFLD	Zucker rats, genetically obese models	Control diet group HFD Raspberry extract (0.63%) + FOSs (3%), 12 weeks	↓body weight gain, ↓body fat, ↓liver mass, ↓FFA, ↓insulin, ↓AST, ↓ALT, ↓ALP, ↑albumin, ↑collagen type IV, ↓TG, and ↓cholesterol. Hepatic oxidative stress and fibrosis indicators: ↓MDA, ↓collagen (measured as hydroxyproline content, ↑GSH/GSSG, ↑*shp*, ↑*fxr*, ↓*ahr*, ↓*cyp7b1*, and ↓*cyp7a1*.
[126]	*Lonicera caerulea* polyphenols (LCP)/NASH	C57BL/6N mice	Normal diet Normal diet + LCP (1%) HFD group HFD + carbon tetrachloride (CCL_4_) HFD + CCL_4_ + LCP (0.5%), HFD + CCL_4_ + LCP (1%), 30 days, CCL_4_ (0.05 mL/BW) to each group every 3 days till day 45 (NASH induction)	Improved histopathological features, ↓GOT, ↓GGT, ↓TG, ↓GPT, ↓G-CSF, ↓KC, ↓TNF-α, ↓IL-1β, ↓IL-1α, ↓IL-2, ↓IL-3, ↓IL-4, ↓IL-5, ↓IL-6, ↓IL-10, ↓IL-13, ↓IL-17, ↓MCP-1, ↓IFN-λ, ↓IL-12(p70), ↓eotaxin, ↓GMCSF, ↓MIP-1α, ↓MIP-1β, and ↓RANTES. Recovered SOD with 1% LCP. Recovered mnSOD and Nrf2 (dose-dependent). ↓TBARS (dose-dependent).
[127]	*Lonicera caerulea* polyphenols (LCBP)/NAFLD	C57BL/6N mice	Normal diet Normal diet + LCBP (1%) HFD group HFD + LCBP 0.5%, or HFD + LCBP 1%, 45 days	↓IL-2, ↓IL-6, ↓MCP-1, ↓TNF-α, ↓endotoxin, ↓*Firmicutes*, ↑*Bacteroidetes*, ↑*Proteobacteria*, ↑*Verrucomicrobia* (with 1%), and ↓*Firmicutes* to *Bacteroidetes* (dose-dependent manner)
[128]	Blue honeysuckle berry extract (BHE)/NAFLD	C57BL/6N mice	Normal diet group HFD group HFD + BHE (0.5%) HFD + BHE (1%), 45 days	↓intra-abdominal fat, ↓TG, ↓glucose, ↓insulin, ↓HOMA-IR (dose-dependent). ↓lipid peroxidation (TBARS), ↑Nrf2, ↑HO-1, and ↑MnSOD.
[129]	Honeyberry extract (HBE) NAFLD	Imprinting control region (ICR) male mice	Normal control diet group HFD group HFD + BHE (0.5%) HFD + BHE (1%), 6 weeks	↓body mass, serum ↓leptin, ↓TC, ↓TG, ↓AST, ↓ALT, ↓NO, ↓MDA, ↓adipocyte size, ↑SOD, ↓PPARγ, ↑GPx, ↑CAT, ↓SREBP-1c, ↓C/EBPα, ↓FAS, ↑CPT-1, ↑PPARα. ↑p-AMPK, ↑p-ACC (dose-dependent)
		HepG2	FFA (1 mM) + HBE (250, 500, and 1000 µg/mL), 24 h	↓lipid accumulation, ↓TG, ↓SREBP-1c, ↓C/EBPα, ↓PPARγ, ↓FAS, ↑CPT-1, ↑PPARα (dose-dependent), ↑p-AMPK, and ↑p-ACC.
[130]	Saskatoon berry powder (SBP)/Steatosis and insulin resistance	C57 BL/6J mice	Control diet group HFHS group HFHS + SBP/kg (5%) and HFHS + C3G/kg (45.2 mg) W/W, 10 weeks	↓fasting plasma glucose, ↓TG, ↓fasting plasma cholesterol, ↓insulin, ↓HOMA-IR, ↓ALT, ↓PNPLA3, ↓PAI-1, ↓MCP-1, ↓TNF-α, ↓NOX2, ↓CHOP, ↓NOX4, ↓MCP-1, and ↓TLR4. Comparable results: SBP (5%) and equivalent dose of C3G (45.2 mg).
[131]	*Schisandra chinensis* (SC) extract/NAFLD	C57BL/6J mice	ER stress development: SC extract (50 and 100 mg/kg BW), 2 days, + DMSO or tunicamycin (1 mg/kg BW) IP, 24 h HFD obese mice Control group HFD group HFD + SC extract group (100 mg/kg/day) HFD and SC extract group (300 mg/kg/day), 16 weeks	↓GRP78, ↓CHOP, ↓TG, and ↓XBP-1c, dose-dependent effect in tunicamycin-injected mice Liver of obese mice: ↓IL-6, ↓TNF-α, and ↓MCP-1.
		HepG2 cells	Tunicamycin- or palmitate-treated + SC extract (10, 50, and 100 μg/mL), 16 h + tunicamycin (2 μg/mL), 6 h or palmitate (400 μM), 24 h	Tunicamycin- or palmitate-treated HepG2: ↓GRP78, ↓CHOP, ↓TG, and ↓XBP-1c. palmitate-treated HepG2: ↓IL-6, ↓TNF-α, and ↓MCP-1. ↓FAS, ↓SCD1, and ↓ACC1.
[132]	*Schisandra chinensis* compound Gomisin J/NAFLD	HepG2 cells	Compound pretreatment (10, 20, and 40 μM), 1 h + OA−BSA complex (0.5 mM), 24 h	↓SREBP-1c, ↓FAS, ↓ACC, ↓HMGCR, ↓DGAT1, ↑PPARα, ↑PPARδ, ↑PGC-1α, ↑ACOX1, ↑CPT-1, ↑UCP2, ↓fetuin-A, ↓TNF-α, ↓MCP-1, ↓NFKB1 ↓NFKB2, ↓TG, ↑p-AMPK, ↑p-ACC, ↑p-CaMKIIβ, and ↑p-LKB1.
[133]	*Schisandra chinensis* berry ethanolic (SCE, 70%) extract/Steatosis	C57BL/6 J mice	Western diet Western diet + SCE (1% *w*/*w*), 12 weeks	↓body weight, ↓liver weight, ↓SREBP-1c, ↓FAS, ↓PPARγ, ↓SCD-1, ↓ACC-1, ↓total acetyl lysine expression, and ↓histone H3 lysine 9 acetylation.
		HepG2	Oleic acid (0.5 mM) + SCE (100, 200 μg/mL), 24 h	↓total acetyl lysine expression. ↓histone H3 lysine 9 acetylation. Inhibited total HAT.
[134]	Extract of dried seabuckthorn leaves (SL) and Flavonoid glycosides extract (SLG)/Steatosis and metabolic disturbances	C57 BL/6J mice	Normal diet group HFD diet group HFD, HFD + SL (1.8% *w*/*w*), HFD + SLG (0.04% *w*/*w*), 12 weeks	↓body weight gain, ↓lipogenesis, ↑energy expenditure, ↑CPT1-*a*, ↓GOT, ↓GPT, ↓FAS, ↓ME, ↓PAP, ↓SREBP-1c, ↑fecal cholesterol, ↑fecal TG, ↑fecal fatty acids, ↑ABCG5, ↑ABCG8, ↓insulin, ↓HOMA-IR, ↓G6Pase, ↓PEPCK, ↑IRS2, ↓GIP, ↓resistin, ↓leptin, ↓TNF-α, ↓IL-1 , ↓IL-6, and ↓PAI-1.
[135]	Sea buckthorn pulp oil (SBPO), sea buckthorn seed oil (SBSO)/NAFLD	male C57BL/6J mice	HFD + SBPO or HFD + SBSO, 12 weeks	Serum and hepatic: ↓TG, ↓LDL-C, ↓hepatic histopathological score, and ↓perirenal fat buildup. Modulated gut microbiota.

ABCG5/8: ATP binding cassette subfamily G member 5/8, α-SMA: alpha smooth muscle actin, ApoA-1: apolipoprotein A1, ACC: acetyl-coenzyme A carboxylase, ACOX1: acyl-coenzyme A oxidase 1, ahr: aryl hydrocarbon receptor, AdipoR2: adiponectin receptor 2, ALT: alanine aminotransferase, Akt: serine/threonine kinase, ap2: adiponectin receptor 2, AST, aspartate transaminase, AMPK: 5′ AMP-activated protein kinase, ANGPTL4: angiopoietin-like 4, ATGL: adipose triglyceride lipase, ASC: apoptosis-associated speck-like protein containing a CARD, BSA: bovine serum albumin, Bax/Bcl-2: Bcl-2 associated X, apoptosis regulator/B-cell lymphoma 2 protein, CCL_4_: carbon tetrachloride, C/EBPα: CCAAT/enhancer-binding protein alpha, C3Glu: cyanidin-3-glucoside, CAT: catalase, CCL2: chemokine ligand 2, Ccr2: C-C chemokine receptor type 2, Cd11c: cluster of differentiation 11c, CD36: cluster of differentiation, Cdkn1a: Cyclin-dependent kinase inhibitor 1A (P21), CHOP: CCAAT-enhancer-binding protein homologous protein, Col1a1: collagen type 1 alpha 1 chain, COX-2: cyclooxygenase-2, CPT-1α: carnitinepalmitoyltransferase-1 alpha, JNK: c-Jun N-terminal kinase, Cxcl14: chemokine (C-X-C motif) ligand 14, Cyp2c: cytochrome P450, family 2, subfamily c, CYP2E1: cytochrome P450 2E1, cyp7a/b1: 25-hydroxycholesterol 7 alpha/beta-hydroxylase, DGAT1: diglyceride acyltransferase 1, Ehhdah: enoyl-CoA hydratase, 3-hydroxyacyl-CoA dehydrogenase, EMT: epithelial–mesenchymal transition, FAS: fatty acid synthase, FFA: free fatty acids, fxr: farnesoid X receptor, FASN: fatty acid synthase, GR: glutathione reductase, G6Pase: glucose-6-phosphatase, GPAT-1: glycerol-3-phosphate acyltransferase 1, Gclc: glutamate–cysteine ligase catalytic subunit, Gclm: glutamate–cysteine ligase modifier subunit, GIP: incretin hormone gastric inhibitory polypeptide, GMCSF: granulocyte-macrophage colony-stimulating factor, GOT: glutamic oxaloacetic transaminase, GPT: glutamic pyruvic transaminase, GPx, glutathione peroxidase, GRP78: glucose-regulated protein 78, GST: glutathione S-transferase, GSH: glutathione, GSSG: oxidized glutathione, HAT: histone acetyltransferase, HDL-C: high-density lipoprotein cholesterol, HFCD: high fat cholesterol diet, HFFD: high-fat high-fructose diet, HFHS: high fat-high sucrose, HMGCR: 3-hydroxy-3-methyl-glutaryl-CoA reductase, HO-1: heme oxygenase-1, HOMA-IR: homeostasis model assessment-estimated insulin resistance, hs-CRP: high-sensitivity C-reactive protein, Hsd17b6: hydroxysteroid (17-beta) dehydrogenase 6, Igfbp2: insulin-like growth factor binding protein 2, iNOS: inducible nitric oxide synthase, IL: interleukin, IP: intraperitoneal, IRS2: insulin receptor substrate 2, JAK1: Janus kinase 1, KC: keratinocyte-derived cytokine, Keap-1: kelch like ECH associated protein-1, LDH: lactate dehydrogenase, LAMP: lysosome-associated membrane proteins, Lcn2: lipocalin 2, LDL-C: low-density lipoprotein cholesterol, LDLR: LDL receptor, Lepr: leptin receptor, LPL: lipoprotein lipase, LXRα: liver X receptor-alpha, M3G: malvidin-3-*O*-glucoside, M3Ga: malvidin-3-*O*-galactoside, MMP13: matrix metallopeptidase 13, MCD: methionine and choline-deficiency diet, MCP-1: monocyte chemotactic protein, MDA: malondialdehyde, ME: malic enzyme, MAPK: mitogen-activated protein kinases, MIP-1α/β: macrophage inflammatory protein-1alpha/beta, Mogat1: monoacylglycerol O-acyltransferase 1, MnSOD: manganese-dependent superoxide dismutase, MyD88: myeloid differentiation primary response 88, NLRP: nod-like receptor protein, NFKB1 and -B2: nuclear factorkappa-B1 and -B2, NO: nitric oxide, NOS2: nitric oxide synthase 2, Notch1: Neurogenic locus notch homolog protein 1, NEFAs: non-esterified fatty acids, NASH: nonalcoholic steatohepatitis, NOX: NADPH oxidase, NQO1: NAD(P)H quinone oxidoreductase 1, OA: oleic acid, OVX: ovariectomized, p-CaMKIIβ: phosphorylated calcium/calmodulin-dependent protein kinase type II beta, p-LKB1: phosphorylated liver kinase B1, PAI-1: plasminogen activator inhibitor-1, PAP: phosphatidate phosphohydrolase, PARP: poly (ADP-ribose) polymerase, PON3: paraoxonase 3, PEPCK: phosphoenolpyruvate carboxykinase, p-ACC: phospho-acetyl-CoA carboxylase, PGC-1α: proliferator-activated receptor gamma coactivator 1-alpha, Plin4: perilipin 4, PNPLA-3: patatin-like phospholipase domain-containing protein 3, PCNA: proliferating cell nuclear antigen, PPAR-α: peroxisome proliferator-activated receptor α, PPARγ: peroxisome proliferator-activated receptor gamma, pSMAD2: phospho smad 2, p-ERK: phosphorylation of extracellular signal-related kinase, RANTES: regulated on activation, normal T-cell expressed and secreted, ROS: reactive oxygen species, S100a10: S100 calcium binding protein A10 (calpactin), Saa: acute-phase inflammatory proteins serum amyloid A, Saa1: serum amyloid A 1, Saa2: serum amyloid A 2, SCD1: stearoyl-CoA desaturase 1, SCFAs: short chain fatty acids, SD: Sprague Dawley, shp: small heterodimer partner, smile: smile small heterodimer partner interacting leucine zipper protein, SHP: short heterodimer partner and small heterodimer partner, SIRT1: sirtuin1, SOD: superoxide dismutase, SiRNA: small interfering RNA, SREBP-1c: sterol regulatory element binding protein-1c, STAT3: signal transducer and activator of transcription 3, TAA: thioacetamide, TBARS: thiobarbituric acid reactive substances, TBHP: tert-butyl hydroperoxide, TG: triglyceride, TFEG: transcription factor EG, TEAC: trolox equivalent antioxidant capacity, TGFβ1: transforming growth factor beta 1, TLR: toll-like receptor, TNF-α: tumor necrosis factor alpha, TIMP: tissue inhibitor of metalloproteinase-1, TC: total cholesterol, Tubb2a: tubulin, beta 2A class IIA, Tubb6: tubulin, beta 6 class V, Txnip: thioredoxin-interacting protein, γ-GCL: gamma-glutamyl cysteine ligase, UCP2: uncoupling protein 2, XBP-1: X-box-binding protein-1, ZO-1: zonula occludens-1. ↑: increase, ↓: decrease.

### 5.11. Acerola 

Acerola (*Malpighia emarginata* DC) is a member of the Malpighiaceae family and is also known as the Barbados cherry or West Indian cherry. It is a tropical plant that grows from South Texas to Mexico, Northern South America, Central America, and the Caribbean, and has recently been introduced in subtropical regions worldwide, including India [136].

The most prevalent type of polymers are polysaccharides, which offer several health-enhancing properties [137]. Hu et al. [110] first showed that repeated administration of acerola polysaccharides can protect against HFD-induced NAFLD by inhibiting hyperlipidemia and hepatic lipid deposition, lowering oxidative stress and inflammation, and enhancing mitochondrial function [110] (Table 1). Future studies should focus on the biological properties of this plant using both in vitro and in vivo models.

### 5.12. Cranberry 

Cranberries (*Vaccinium macrocarpon*) are rich in phenolic compounds and belong to the family Ericacea. In addition to anthocyanins, proanthocyanins, phenolic acids, terpenes, and flavonols have also been reported in cranberries [138]. The beneficial effects of cranberries have been reported against oxidative stress, diabetes, obesity, cardiovascular diseases, *Helicobacter pylori* infections, cancers, urinary tract inflammation, and kidney diseases [139]. A few studies have evaluated the effects of cranberries on NAFLD models.

Glisan et al. [111] reported that dietary supplementation with cranberry extract mitigated the progression of NAFLD in an HFD-induced obese mouse model. Cranberry extract reduced NAFLD severity by 33% at the histological level and reduced lipid droplet size by 29%. Moreover, the NAFLD and hepatic inflammation-reducing effects of cranberry extract were found to be related to the mitigation of toll-like receptor 4/NF-κB related signaling [111] (Table 1). Another study reported the anti-fibrotic effects of cranberry nutraceuticals in a high-fat cholesterol diet-induced NAFLD model. Cranberry nutraceuticals were specifically evaluated to improve insulin sensitivity and modulate Nrf2 expression [112]. Similarly, another study revealed the protective effects of cranberry against NAFLD by lowering oxidative stress and serum TG accumulation in HFD-fed mice [113] (Table 1). These studies demonstrated the preventive and therapeutic effects of cranberries in NAFLD models.

#### Clinical Study 

Mohammadshahi et al. [140] evaluated the effects of cranberry supplementation (288 mg tablet) on antioxidant, inflammatory, and apoptotic biomarkers in 41 patients with NAFLD for 12 weeks. The cranberry group showed a marked improvement in the total antioxidant capacity (TAC). However, no significant differences were reported for serum levels of malondialdehyde (MDA), CK-18 M30, chemokine ligand 2, and TNF-α. This study showed that cranberry supplementation can improve serum TAC levels; however, further studies are warranted to investigate its anti-inflammatory and antioxidant effects [140]. 

### 5.13. Mangosteen 

Mangosteen (*Garcinia mangostana*) is a member of the Clusiaceae family, famous as purple mangosteen. This tropical cultivable plant produces edible fruits and is found in Southeast Asia, Africa, and Central America. The peel extracts of *G. mangostana* unveiled hepatoprotective activity against thioacetamide (TAA)-induced liver cirrhosis in rats [114]. Reports show that the hepatotoxic effects of TAA can be observed when cytochrome P450 oxidases shift to deadly intermediates, followed by an increase in ROS, MDA, and proinflammatory cytokines [141]. The protective effects of *G. mangostana* peel extracts against TAA-induced hepatotoxicity could be because of their ability to suppress hepatocyte proliferation, reduce lipid peroxidation and oxidative stress, and possess antioxidant potential. Future research should focus on the identification of phytoconstituents with hepatoprotective actions and their effects on different signaling pathways [114]. Another study evaluated the effects of *G. mangostana* on apoptosis and autophagy in an HFD-induced NAFLD-NASH mouse model. *Garcinia* treatment suppressed hepatocyte apoptosis and enhanced autophagy, in addition to improving other liver functions [115] (Table 1). However, further studies are required to test the biological potential of this fruit. 

### 5.14. Lingonberry

Lingonberry (*Vaccinium vitis-idaea* L.) is a member of the Ericaceae family. Lingonberries are small, perennial shrubs found throughout Asia, Greenland, Iceland, Scandinavia, North America, and Europe [142]. The potential health benefits of lingonberries include their antioxidant, anti-inflammatory, and anticancer activities, as well as protection against neurodegenerative disorders [143]. 

Previous research has reported that lingonberries modulate gut microbiota and prevent low-grade inflammation in HFD-fed animals [144]. Further research by this team demonstrated that animals fed an HFD and lingonberry powder (air-dried) were protected from adverse changes in the liver by preventing the effects of the HFD on genes involved in lipid or glucose metabolic processes, inflammatory or immune response, cell migration, or cell cycle changes [116] (Table 1). The hepatoprotective effects of lingonberry supplementation were attributed to activation of the Nrf2 signaling pathway [117]. Expression of the hepatic neurogenic locus notch homolog protein 1 (Notch1) receptor has been reported in both animal models and human patients with NAFLD [145]. In a follow-up study, the research group discovered that the protective effects of lingonberry against HFD-induced fatty liver were partly owing to the inhibition of Notch signaling [118]. These studies suggest that lingonberries may be a good option for inclusion in regular diets. However, further clinical studies are required to reach definitive conclusions.

### 5.15. Maoberry 

Maoberry (*Antidesma bunius*) belongs to the family Stilaginaceae and is commonly grown in the warm climates of tropical Asia, Australia, and Africa. Maoberries are reported to have many health benefits owing to the presence of polyphenolic compounds, particularly anthocyanins [146]. 

Ngamlerst et al. [119] have demonstrated the protective effects of macrophages in an HFD-fed rat model. Maoberry supplementation reduced fat droplet size and downregulated the expression of key enzymes involved in lipid production. Additionally, the expression of inflammation-related genes was also downregulated [119] (Table 1). Thus, the biological properties of maoberry have been sparsely evaluated, and more studies are required in the near future. 

### 5.16. Maqui 

Maqui (*Aristotelia chilensis*), also referred to as Chilean wineberry, is a natural berry that has recently attracted increased attention from around the world owing to its potential antioxidant, anti-inflammatory, and protective effects on cardiovascular health and regulation of blood sugar metabolism [147,148].

In a previous study, the induction of browning in subcutaneous white adipose tissue and amelioration of IR were demonstrated by maqui in an HFD-induced obese mouse model [149]. In a subsequent study, the same research group evaluated the effects of lyophilized maqui berries on reducing lipogenesis in an HFD-induced mouse model by upregulating the expression of the nuclear receptor family’s corepressor small heterodimer partner interacting with leucine zipper protein, commonly known as SMILE, and downregulating lipogenic target genes and fatty acid oxidation genes [120] (Table 1). This study supports the potential use of maqui berries for the treatment of obesity and MetS. However, further in vitro and in vivo studies are required to confirm these findings. 

### 5.17. Raspberry 

Raspberries belong to the family Rosaceae and are represented by the genus *Rubus idaeus* [150]. Raspberries are reported to contain important active compounds such as phenolics, organic acids, anthocyanins, and minerals. Raspberries are known to possess antioxidant, anti-inflammatory, antiobesity, anticancer, antimicrobial, and oral health application properties [151,152]. The two major phytoconstituents of raspberries are anthocyanins and ellagitannins. Raspberry ketone is one of the major aromatic compounds in raspberries and is generally used as a flavoring agent in the food industry, as well as for fragrance in the cosmetics industry [153]. The role of raspberry ketones in protecting the liver has also been reported [154]. 

**Figure 7:**
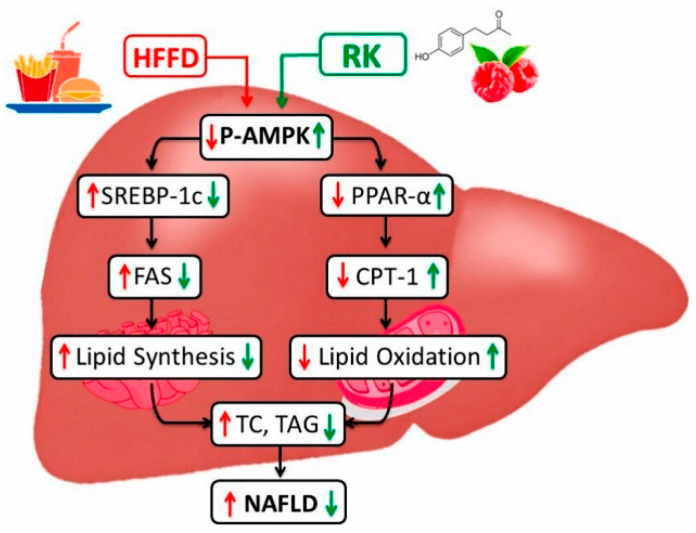
The protective effect of raspberry ketone (RK) on high-fat high fructose diet-induced fatty livers in rats. The diagram illustrates the mechanism behind the protective action of RK. P-AMPK: phosphorylated AMP-activated protein kinase, SREBP-1c: sterol regulatory element-binding protein-1c, PPAR-α: peroxisome proliferator-activated receptor alpha, FAS: fatty acid synthase, CPT-1: carnitine palmitoyltransferase-1, TC: total cholesterol, TAG: triacylglycerol, NAFLD: nonalcoholic fatty liver disease. ↑: increase, ↓: decrease. Reproduced with kind permission from Ref. [122]. Copyright 2023 Elsevier.

Wang et al. [121] have documented the use of raspberry ketone as a natural intervention in an animal model of NASH. This study reports the dual effects on liver protection and fat reduction. The authors suggested that the protective effects of raspberry ketone were because of their ability to reduce hepatic inflammation, alleviate dyslipidemia, improve antioxidant capacity, and reverse IR [121]. Mechanistically, a recent study documented that raspberry ketone ameliorates NAFLD in vivo by activating the AMPK signaling pathway (Figure 7). The authors advocated the use of this treatment approach over a long-term calorie restriction routine, which may be affected by poor compliance [122] (Table 1). 

More recently, the protective effects of raspberry ketones against hepatic fibrosis have been reported in cellular and animal models. Raspberry ketones inhibit inflammation, extracellular matrix accumulation, and epithelial–mesenchymal transition. In a previous study, double activation of the farnesoid X receptor (FXR)/PGC-1α was shown to be a target for raspberry ketones [123]. 

Based on the notion that dietary combination would increase the beneficial effects of berries, Fotschki et al. [124] combined raspberry polyphenolic extract with non-digestible saccharide, fructooligosaccharides, or pectin and evaluated the effects of different treatments on the NAFLD rat model. This study evaluated its effects on hepatic lipid metabolism, inflammation, and cecal microbial fermentation. In both treatments, the raspberry extract containing fructooligosaccharides was the most effective. This study suggests that dietary fiber supplementation results in higher raspberry activity in rats fed an obesogenic diet. The combined treatment effectively reduced the concentration of SCFAs in the caecum [124]. Inspired by the results of their previous study, the authors evaluated the effects of raspberry extract and fructooligosaccharides against advanced-stage NAFLD in Zucker rats with genetic susceptibility to obesity. The treatment improved antioxidant status and inflammation, mitigated anomalies related to glucose and lipid metabolism, and reduced indicators of liver fibrosis development (Table 1). The observed favorable effects were plausible because of the activation of the FXR-linked mechanism documented for bile acid homeostasis and hepatic aryl hydrocarbon receptor with multifaceted physiological functions. However, clinical studies are warranted to ascertain the full potential of raspberry extract and prebiotics in humans [125]. 

#### Clinical Study 

A recent systematic review and meta-analysis of 10 RCTs with 355 participants reported that raspberry consumption had not impacted anthropometric indices and liver function tests. This study has some limitations. The study participants were from a variety of target categories, including those at risk of MetS, prediabetes, type 2 diabetes, and menopause symptoms, and healthy adults, which may have influenced the study results. Furthermore, most of the included trials were found to have a high overall risk of bias. Another limitation is that most of the included trials did not indicate the power of their investigations; thus, their findings should be interpreted with caution. To draw definite conclusions, well-designed RCTs with a large sample pool are required in the near future [155]. 

### 5.18. Honeyberry 

Honeyberry (*Lonicera caerulea* L.) is also known as blue honeysuckle berry, haskup, haskap, or sweet berry honeysuckle. The plant contains phenolic acids, flavonoids, iridoids, triterpenoids, fatty acids, and organic acids, and has been reported to exhibit many health-promoting activities, including anti-diabetic, antioxidant, anti-inflammatory, antimicrobial, antitumor, hepatoprotective, lung protective, anti-obesity, and cardioprotective effects [156]. 

The attenuating effects of this underutilized berry on NASH have been previously reported in vivo. For instance, Wu et al. [126] reported that the treatment of HFD- and carbon-tetrachloride-treated NASH mice resulted in reduced lipid peroxidation, IR, and inflammation by upregulating the expression of Nrf2 and MnSOD, suppressing proinflammatory cytokine production, and downregulating forkhead box protein O1 and heme oxygenase-1 in the livers of mice [126] (Table 1). In another study, researchers reported the modulating effects of honeyberry phenolic compounds on the gut microbiota in HFD-induced NAFLD mice. Forty-five days’ treatment lowered the Firmicutes to Bacteroidetes ratio and increased the abundance of Bacteroides and Parabacteroides. As reported in a previous study, polyphenol supplementation significantly decreases proinflammatory cytokine levels [127]. Another study demonstrated the mitigating effects of honeyberry extract on oxidative stress markers by upregulating the Nrf2-mediated pathway in HFD-fed mice. These positive effects were dose-dependent [128] (Table 1). These studies suggest that the mitigating effects of honeyberry extracts could be because of their combined antioxidant and anti-inflammatory activities against NAFLD. 

We assessed the beneficial effects of honeyberry extract on FFA-induced HepG2 cells and HFD-fed mice by modulating AMPK and ACC signaling. The beneficial effects of honeyberry may be attributed to the presence of the anthocyanin cyanidin 3-*O*-glucoside (C3G), as investigated using high-performance liquid chromatography with diode array detection [129]. These studies demonstrated the beneficial effects of honeyberry extract on oxidative stress markers, insulin sensitivity, and obesity-associated markers. Further research, along with clinical studies, should compare geographical and seasonal variants and their effects on NAFLD and NASH models. 

### 5.19. Saskatoon Berry

Saskatoon berry (*Amelanchier alnifolia* Nutt.) is a tall shrub belonging to the family Rosacea that is native to North America, the northwestern states of the United States, and the prairies of Canada [157]. The major phenolic components of saskatoon berries are flavonoids (quercetin, rutin, hyperoside, and avicularin), anthocyanins, and proanthocyanins [158]. Cyanidin-based anthocyanins are major anthocyanins include C3G, cyanidin 3-*O*-galactoside, cyanidin 3-*O*-xyloside, and cyanidin 3-*O*-arabinoside [159,160]. Previous studies have suggested that saskatoon leaves and berries have antioxidant, anti-inflammatory, anti-diabetic, antitumor, and gut microbiota-modulating activities [157,158]. 

Few studies have reported the ameliorating effects of saskatoon berries on NAFLD. Zhao et al. [130] evaluated the effects of saskatoon berry powder and C3G in a high-fat, high-sucrose-stimulated hepatic steatosis animal model. Both berry extract and C3G exhibited similar effects on liver steatosis, hyperlipidemia, hyperglycemia, IR, inflammation, and oxidative stress. The beneficial effects of berry extract can be attributed to the presence of the active compound, C3G. The results suggest that supplementation with saskatoon berry extract and its active components mitigates diet-induced hepatic steatosis, IR, and inflammation. Future research should focus on the precise mechanisms underlying these beneficial outcomes and conduct well-designed clinical studies [130] (Table 1). 

### 5.20. Schisandra Berry

As per Chinese pharmacopeia, *Schisandra chinensis* is also known as Bei Wu Wei Zi and documented to have many health-promoting properties such as antioxidant, cardioprotective, antibacterial, anticancer, anti-inflammatory, and hepatoprotective [161]. Its cultivation dates back to Korea, China, Japan, and Russia [162]. 

**Figure 8:**
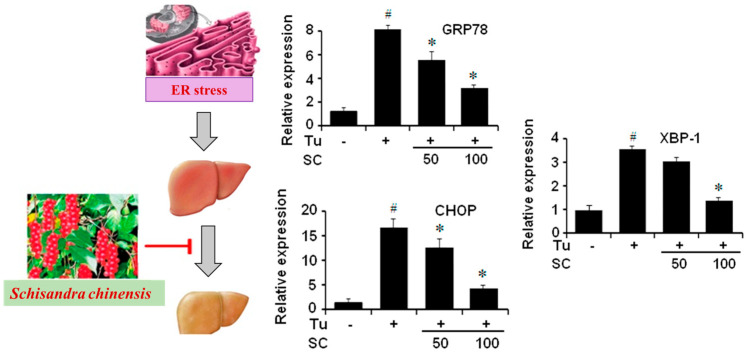
The protective effects of *Schisandra chinensis* (SC) extract in tunicamycin-injected mice in vivo. Different doses of SC extract reduced the expression of ER stress markers. ER: endoplasmic reticulum, GRP78: glucose-regulated protein 78, CHOP: C/EBP homolog protein, XBP-1: X-box-binding protein-1. ^#^ represents *p* < 0.05 vs. control without treatment and * represents *p* < 0.05 vs. tunicamycin-treated control. Reproduced (with modifications) with kind permission from Ref. [131]. Copyright 2016 Elsevier.

Long-term endoplasmic reticulum stress has been reported to be a major contributor to NAFLD. Jang et al. [131] established that *S. chinensis* extract has beneficial effects against NAFLD by inhibiting endoplasmic reticulum (ER) stress markers in HepG2 cells and mouse livers stimulated with tunicamycin (Figure 8). The extract ameliorated stress in palmitate-treated HepG2 cells. Supplementation with the extract downregulates the expression of lipogenic genes in the liver [131] (Table 1). 

*S. chinensis* contains many bioactive components including gomisin N, gomisin J, schisandrol A, schisandrol B, tigloylgomisin H, angeloylgomisin H, and schisandrin C, B, and A. The effect of gomisin J on lipid metabolism has been reported to reduce oleic acid-induced lipogenesis and induce lipolysis in HepG2 cells by increasing the activation of AMPK, kinase B1 (liver), and calcium/calmodulin-dependent protein kinase II, as well as by inhibiting fetuin-A [132]. Fetuin-A is a secretory glycoprotein and a known biomarker of chronic inflammatory diseases. Chung et al. [133] reported the mitigating effects of this berry on epigenetic regulation and mechanisms responsible for the development of hepatic steatosis. The ethanolic extract of the berries exhibited alleviating effects owing to its hypolipidemic activity through the inhibition of histone acetyltransferase. The extract attenuated the acetylation of total lysine and lysine 9 (K9) in the tail of histone H3. The acetylation at these positions is strongly associated with gene expression [133]. Future studies should evaluate the effects of the bioactive components present in berries on acetylation inhibition and the development of hepatic steatosis. 

### 5.21. Sea Buckthorn

Sea buckthorn (*Hippophae rhamnoides* L.) is a plant with health benefits that is cultivated in Asia, Europe, and Canada [163]. Members of the Elaeagnaceae family thrive in deep sandy loam soil that is well-drained and rich in organic material. Berries, bark, and leaves are reported to contain many active substances, as well as various health-promoting activities, such as anti-atherogenic, antioxidant, anticancer, hepatoprotective, cytoprotective, immunomodulatory, and antimicrobial activities [163,164]. 

The use of different parts of this plant in the treatment of NAFLD has also been reported. A previous study used sea buckthorn leaf extract and flavonoid glycoside extract to treat hepatic steatosis and other metabolic disturbances in HFD-fed animals. Both supplements improved hepatic steatosis by enhancing liver fatty acid oxidation and suppressing lipogenesis and lipid absorption in the liver. Improved insulin sensitivity, increased energy expenditure, and suppressed glucogenic enzyme activity have been reported. This indicates that sea buckthorn leaf extract, especially its flavonoid glycosides, ameliorated hepatic steatosis, inflammation, dyslipidemia, IR, and obesity [134] (Table 1). 

Recently, the effectiveness of sea buckthorn pulp and seed oils was evaluated in NAFLD mice. The two extracts exhibit different bioactive constituent profiles. Supplementation with sea buckthorn pulp oil resulted in reduced adipocyte sizes, reduced visceral fat buildup, lowered levels of TG and LDL-C in the serum and liver, and lower damage to the top liver cells. The dietary intervention also modulated the gut microbiota compared to peanut oil; higher levels were reported for *Lactobacillus*, *Roseburia*, and *Oscillibacter.* Dietary intervention with sea buckthorn pulp oil increased microbial community diversity, whereas sea buckthorn seed oil increased species richness. Overall, sea buckthorn seed oil exhibited similar or weaker effects than sea buckthorn pulp oil. The differences in their effects on metabolic parameters may be attributed to the differences in fatty acid profiles and other metabolites [135]. These studies indicate that sea buckthorn plant parts are rich in bioactive substances and can be utilized for the treatment of NAFLD and other metabolic disorders through the modulation of gut microbiota. 

#### Clinical Study

Gao et al. [165] reported the effects of sea buckthorn on patients with NAFLD recruited from a Chinese hospital. For treatment, 1.5 g of sea buckthorn was administered to 48 patients with NAFLD three times a day and 46 controls for three months. Sea buckthorn treatment resulted in significantly lower levels of ALT, lactate dehydrogenase (LDL)-C, type IV collagen, and procollagen III. Furthermore, as shown by FibroSca, the liver stiffness measurement of the treated subjects was observed to be significantly lower, while the computed tomography scan demonstrated an increased liver/spleen ratio compared to that in the controls and at baseline. The results showed significant differences between the groups [165]. 

The limitations of this study include the fact that NAFLD was diagnosed solely through ultrasonography, with no pathological confirmation, and the duration was short. Another limitation is that the function of synergism, such as a restricted diet or exercise program, cannot be ignored when evaluating the beneficial effects of sea buckthorn. Furthermore, hypoglycemic medicines have been used in some patients with diabetes, and chemical metformin alone can be used to treat NAFLD. More well-planned, long-duration RCT trials are required to ascertain the conclusions regarding these berries. 

## 6. Mechanisms 

The common mechanisms by which berries mitigate NAFLD involve: (1) decreasing lipid buildup and preserving lipid homeostasis, (2) alleviating oxidative stress through the enhancement of antioxidant levels, (3) inhibition of inflammatory pathways, (4) promotion of β-oxidation to mitigate mitochondrial dysfunction, and (5) activation of several signaling pathways.

However, some different mechanisms have also been reported by a few studies, suggesting the importance of other mechanisms in mitigating NAFLD. For example, anthocyanin M3G isolated from blueberries, besides mitigating ROS, promoted biogenesis and function of lysosomes through TFEB regulatory protein by translocating from the cytoplasm into the nucleus and attaching to the coordinated lysosomal expression and regulation elements in the cell model [72]. 

Among others, improvement in gut microbiota dysbiosis has been observed as the beneficial effect of phenolic compounds from *Aronia melanocarpa* in LPS-induced liver diseases, anthocyanin from bilberry against the western diet-induced NAFLD mice model, blackberry leaf and fruit extract in HepG2 cells, and phenolic compounds in honeyberry in HFD-induced animals. Of note, an increase in the abundance of *Akkermansia* bacteria was reported in the animal group supplemented with HFD and a high dose of honeyberry (1%) phenolic compounds [127]. Furthermore, treatment with goji berry phenolic compounds along with medium-intensity aerobic exercise showed additional beneficial effects on hepatic inflammation, intestinal barrier, and gut microbiota diversity compared to either phenolic compounds or aerobic exercise, highlighting the importance of physical activity along with medicine for improving NAFLD [49].

Most of the studies evaluated the beneficial effects of berries on downregulating PGC-1α, which acts as a coactivator of genes related to gluconeogenesis; however, Maqui berry supplementation increased SMILE expression, an insulin-inducible corepressor that maintains lipid homeostasis by regulating SREBP1c and LXR activities and also suppresses gluconeogenesis [120]. 

Potential mechanisms for ameliorating hepatic fibrosis include reversing fibrogenesis through regulation of ECM and collagen deposition, reducing inflammation, and promoting the epithelial–mesenchymal transition. Targeted imitation between FXR and PGC-1α by raspberry ketone was reported as a potential mechanism against hepatic fibrosis [123]. Polysaccharides from goji berries suppressed hepatic fibrosis by reducing the transforming growth factor-β/SMAD pathway, alleviating collagen deposition and emphasizing the mechanism that inhibits fibrosis [44,48].

The hypolipidemic effects of ethanolic extracts of *Schisandra chinensis* berry have been reported through the inhibition of histone acetyl transferases in vitro and in vivo. Natural HAT inhibitors present in different berries should be identified and focus of the future research [133]. Another interesting mechanism that prevented the development of NAFLD is the inhibition of ER stress markers by the methanolic extract of *Schisandra chinensis* berry [131]. Future studies are required to assess the beneficial effects of different berries against the development of NAFLD induced through ER stress.

## 7. Conclusions and Future Perspectives

This review summarizes the NAFLD literature and provides a comprehensive account of studies on the beneficial effects of various berries, plant parts, and bioactive compounds against NAFLD and related pathologies in different biological responses (in vitro and in vivo) as well as clinical studies. To the best of our knowledge, this is the first comprehensive study describing the beneficial effects of twenty-one berries on NAFLD markers. Berry’s intervention data clearly demonstrated that NAFLD could be improved through a variety of mechanisms, including maintaining lipid homeostasis by inhibiting or activating key enzymes involved in lipid metabolism, regulating lipid transport, improving liver enzymes and IR, relieving oxidative stress, regulating inflammatory molecules, and activating different signaling pathways. Few studies have reported on the beneficial effects of berries and probiotics, emphasizing the importance of synergy, which should be the focus of future research. Furthermore, addressing gut microbiota dysbiosis has been shown to be a viable treatment approach for managing NAFLD. Hence, further research is required to determine the precise mechanisms through which berries modulate the dysregulation of gut microbiota and their metabolites in NAFLD [166]. The compiled literature on clinical studies shows the ameliorating effects of only a few berries on NAFLD patients through improved TAC and regulation of lipid metabolism markers. However, comparable findings need to be obtained for other berries. Long-term clinical studies targeting gut microbiota should be conducted in the near future. Fecal microbiota transplantation is a recent and clinically promising breakthrough in NAFLD management [167]. Hence, future research should focus on clinical studies, potential risks, and public awareness of fecal microbiota transplantation. Furthermore, fibrosis is considered an important histological determinant; hence, research on the anti-fibrotic effects of any therapeutic molecule in question is regarded as a new aspect of research [168].

Despite the beneficial outcomes of berry intervention in NAFLD in vivo and in clinical studies, some questions need to be answered. Undeniably, preclinical models are essential tools for studying liver disease pathology. However, the disease profile varies greatly across rat models, making it difficult to identify an optimal rodent model that can fully replicate the entire NAFLD spectrum within an acceptable timeframe. Additionally, existing knowledge of age, sex, and hormonal status should facilitate the interpretation of data and their translational potential [169]. Hence, the preclinical beneficial outcomes that are likely to be reproduced in NASH clinical studies remain challenging. To bridge this gap between preclinical and clinical studies, animal studies should utilize openly accessible gene profile data derived from biopsies of healthy controls and patients with NASH to validate animal models [170]. Owing to the invasive procedure and potential for sampling errors, the development of reliable, noninvasive diagnostic techniques capable of detecting the disease in both the initial and advanced phases is a lucrative choice [171]. Furthermore, the construction of preclinical models can also be based on clinical trial outcomes with the goal of using these medications in animal models to produce effects similar to those observed in clinical studies.

Multilineage liver spheroids and organoids are favored over traditional monolayer cell cultures for investigating NASH as these three-dimensional models more accurately reflect the interactions among diverse liver cell types, hepatic morphology, and intracellular communication [172,173]. Incorporating omics methods, such as proteomics and lipidomics, into preclinical studies may yield a thorough comprehension of animal models and enhance their assessment as translatable preclinical models for human NASH. Recently, multiomics (proteomics, lipidomics, and metabolomics) has been used to investigate metabolic changes in patients with NASH [174].

Investigations into extracellular vesicles may offer a potential therapeutic target for NASH treatment, acting as signaling mediators that exacerbate inflammation, leading to lipid accumulation, macrophage activation, hepatic stellate cell activation, and the progression of liver fibrosis. Additionally, targeting the gut–liver axis and gut microbiota, stem cell therapy and genetic approaches warrant further exploration of these emerging strategies as potential treatments for NAFLD [175].

The bioavailability and pharmacokinetics of the tested berries and the identification of the active compounds responsible for their ameliorative effects against NAFLD are crucial factors that need to be considered. Comprehensive research is required to identify and isolate the active components or compounds in berries. Emphasis should be placed on the mechanism of action of berry compounds, either alone or in combination with other compounds and probiotics, to prevent or manage fatty liver. Machine learning approaches should be implemented to optimize various strategies for predicting NAFLD in the general population [176,177]. Overall, for berries and their bioactive components to be as effective against NAFLD, they should be able to ameliorate steatosis, liver inflammation, fibrosis, obesity, glucose metabolism, and IR.
